# Decoding the evolutionary history of ST30 *Staphylococcus aureus*: insights into a potentially silent MSSA bloodstream pathogen

**DOI:** 10.3389/fmicb.2025.1522747

**Published:** 2025-04-09

**Authors:** Matheus Assis Côrtes Esteves, Mariana Fernandes Carvalho, Alice Slotfeldt Viana, Caroline Lopes Martini, Luis Guilherme Araújo Longo, Deborah Nascimento Santos Silva, Adriana Lucia Pires Ferreira, Bernadete Teixeira Ferreira-Carvalho, Paul Joseph Planet, Agnes Marie Sá Figueiredo

**Affiliations:** ^1^Departamento de Microbiologia Médica, Instituto de Microbiologia Paulo de Góes, Universidade Federal do Rio de Janeiro, Rio de Janeiro, RJ, Brazil; ^2^Faculdade de Medicina, Instituto de Educação Médica (IDOMED), Universidade Estácio de Sá, Rio de Janeiro, RJ, Brazil; ^3^Diagnósticos da América S.A., Rio de Janeiro, RJ, Brazil; ^4^Children’s Hospital of Philadelphia, Philadelphia, PA, United States; ^5^Perelman School of Medicine, University of Pennsylvania, Philadelphia, PA, United States; ^6^Programa de Pós-Graduação em Patologia, Faculdade de Medicina, Universidade Federal Fluminense, Rio de Janeiro, RJ, Brazil

**Keywords:** MRSA, *Staphylococcus aureus* evolution, phage-type 80/81, Southwest Pacific clone, toxic-shock syndrome toxin

## Abstract

**Background:**

*Staphylococcus aureus* clonal complex 30 (CC30) is a historically significant pathogen affecting both hospital and community settings. The notable pandemic clones, phage-type 80/81 (PT80/81) and the Southwest Pacific clone (SWP) have spread internationally, contributing to significant morbidity and mortality. Despite their importance, research on the evolution of sequence type (ST) 30 has been limited, often focusing on a small number of strains or specific regions.

**Methods:**

In this study, we analyzed over 500 ST30 genomes from diverse sources, including Brazilian strains sequenced by our team, using genomic, pangenomic, phylogenetic, and time-calibrated phylogenetic analyses.

**Results:**

We traced key evolutionary events, estimating that the specialization of PT80/81 and SWP occurred after a divergence around 1868, forming a group of PT80/81-related strains and another group formed by SWP-related strains. Our findings highlight major events involving gene acquisition and loss, as well as mobile genetic elements (MGE). Notably, PT80/81 lost most *lpl* genes during diversification, which may have restricted the circulation of related strains. Contemporary strains—defined as those that emerged in the 21st century—predominantly cluster within a group divided into three subgroups, including Brazilian strains that acquired a novel pathogenicity island. Also clustering within the contemporary group, most toxic shock syndrome toxin-1 (TSST-1)-producing strains are methicillin-susceptible *S. aureus* (MSSA) that have gained additional virulence traits, including *sea*, which enhance their adaptability and virulence.

**Conclusion:**

Our study revises the evolutionary history of ST30 *S. aureus* uncovering critical pathoadaptive events that may explain its success. Additionally, our findings emphasize a neglected issue: the high prevalence of MSSA in hospital infections, particularly the silent circulation of TSST-1 producing strains, capable of causing severe infections. Robust surveillance studies to monitor these strains are crucial.

## 1 Introduction

A pivotal event in the history of *Staphylococcus aureus* was the global dissemination of penicillin-resistant strains of PT80/81 during the 1950s (Shaffer et al., 1957). These methicillin-susceptible *S. aureus* (MSSA) of clonal complex (CC) 30 and sequence type (ST) 30 were first identified in neonatal infections in Australia in 1953. They rapidly spread worldwide, causing hospital-associated (HA) and community-acquired (CA) infections, including skin infections, pneumonia, and fatal sepsis (Rountree and Beard, 1958; Jessen et al., 1969). After about a decade of widespread prevalence, these strains showed a decline in newborn colonization, and a subsequent decrease in adult infections (Jessen et al., 1969). The pandemic decline coincided with the clinical introduction of methicillin, a semisynthetic penicillinase-resistant β-lactam, in the 1960s (Jevons, 1961).

In the early 2000s, CA methicillin-resistant *S. aureus* (CA-MRSA) strains of the lineage ST30-SCC*mec*IV known as Southwest Pacific clone (SWP) or Oceania Southwest Pacific clone (OSPC) emerged in Oceania (Huygens et al., 2002; Okuma et al., 2002). Both PT80/81 and SWP strains can produce Panton-Valentine leucocidin (PVL), leading to the hypothesis that they share a close evolutionary relationship (Riley et al., 1998; Nimmo et al., 2000; Gordon and Lowy, 2008). After its initial identification in Oceania, ST30-MRSA was reported in CA infections across South America, particularly in Brazil, and Uruguay, as well as in European countries and other continents (Ma et al., 2006; Ribeiro et al., 2007; Rossney et al., 2007; Bartels et al., 2010; Rasigade et al., 2010; Aschbacher et al., 2012; Brauner et al., 2013). Although early reports associated ST30-MRSA primarily with CA infections, strains from these lineages have since become significant HA pathogens in various countries (Silva-Carvalho et al., 2009; McGavin et al., 2012; Antonelli et al., 2019; Doudoulakakis et al., 2022). In certain regions, PVL-positive ST30-MRSA has emerged as the second most common lineage, accounting for 18.2–21.5% of MRSA isolates (Goudarzi et al., 2020; Viana et al., 2021).

Despite the global spread and significance of ST30 strains in staphylococcal infections, few studies have explored the evolution of these important microorganisms using whole genome sequencing (WGS) approaches. Most existing research has relied on a limited number of isolates or has been regionally restricted, and only a few studies focusing on South American countries like Brazil (DeLeo et al., 2011; McAdam et al., 2012; Durand et al., 2018; Di Gregorio et al., 2021; Campbell et al., 2022). The central aim of our study was to investigate the role of mobile genetic elements (MGEs) in the evolution of ST30, particularly in terms of how they drive changes in bacterial virulence and epidemiology. In this study, we applied genomic, pangenomic, phylogenomic, and evolutionary strategies with a comprehensive and representative collection of ST30-MSSA and ST30-MRSA genomes from multiple continents to reassess the evolutionary history of this pathogen through the lens of MGE-driven evolution. Our findings provide fresh insights into the evolution of ST30 strains, emphasizing the critical role of MGEs in the evolutionary divergence that shaped the specialization of these important pathogens. Notably, we identified an MSSA clone capable of silently infecting patients in intensive care units (ICUs), causing invasive and severe infections on a global scale.

## 2 Materials and methods

### 2.1 Genome selection

We selected 441 ST30 genomes from a database of 3,015 ST30 genomes downloaded from NCBI on 06.06.2023, prioritizing regional diversity and the availability of key information such as isolation year, country, clinical origin, and genome quality. Additionally, 63 ST30 sequences from the Sequence Read Archive (SRA, NCBI) were included to incorporate reference strains of PT80/81, such as M1015, M1016, NRS216, and 65–20, and strains of SWP, including WBG10049, TCH60, and 01.7997.S along with 37 genomes from Brazil sequenced by our team (36 ST30 and one ST4279). The ST4279 differed from ST30 by only one nucleotide in the *glpF* gene. Further details on the selection of this collection are available in Supplementary File S1. Supplementary Table S1 provides NCBI accession numbers and other relevant information for the 541 genomes used in this study. Molecular typing of the genomes was performed using MLST, accessible at https://github.com/tseemann/mlst, staphopia-SCCmec (Petit III and Read, 2018) and spaTyper, available at https://github.com/HCGB-IGTP/spaTyper.

### 2.2 Genome sequencing and assembly

Genomic DNA was obtained using the Wizard Genomic Kit (PROMEGA, Madison, WI, USA). Libraries were prepared with the Nextera DNA Flex Library Prep Kit (Illumina, San Diego, CA, USA), and paired-end sequencing was performed on an Illumina MiSeq with 300 cycles. Raw files were trimmed using BBDuk v.38.84, normalization was performed using BBNorm v.38.84 (median coverage of 40x, minimal coverage threshold of 6x, and a K-mer size of 31) and assembled de-novo with Velvet v.1.2.10, all within the Geneious Prime v.2023.2.1 (Biomatters, Auckland, NZ). The completeness of the normalized assemblies, including the preservation of repetitive informative sequences, was validated using QUAST^1^ and Mobile Element Finder v1.0.3 (MobileElementFinder/).^2^

### 2.3 Phylogenetic analysis and annotation

**Figure 1:**
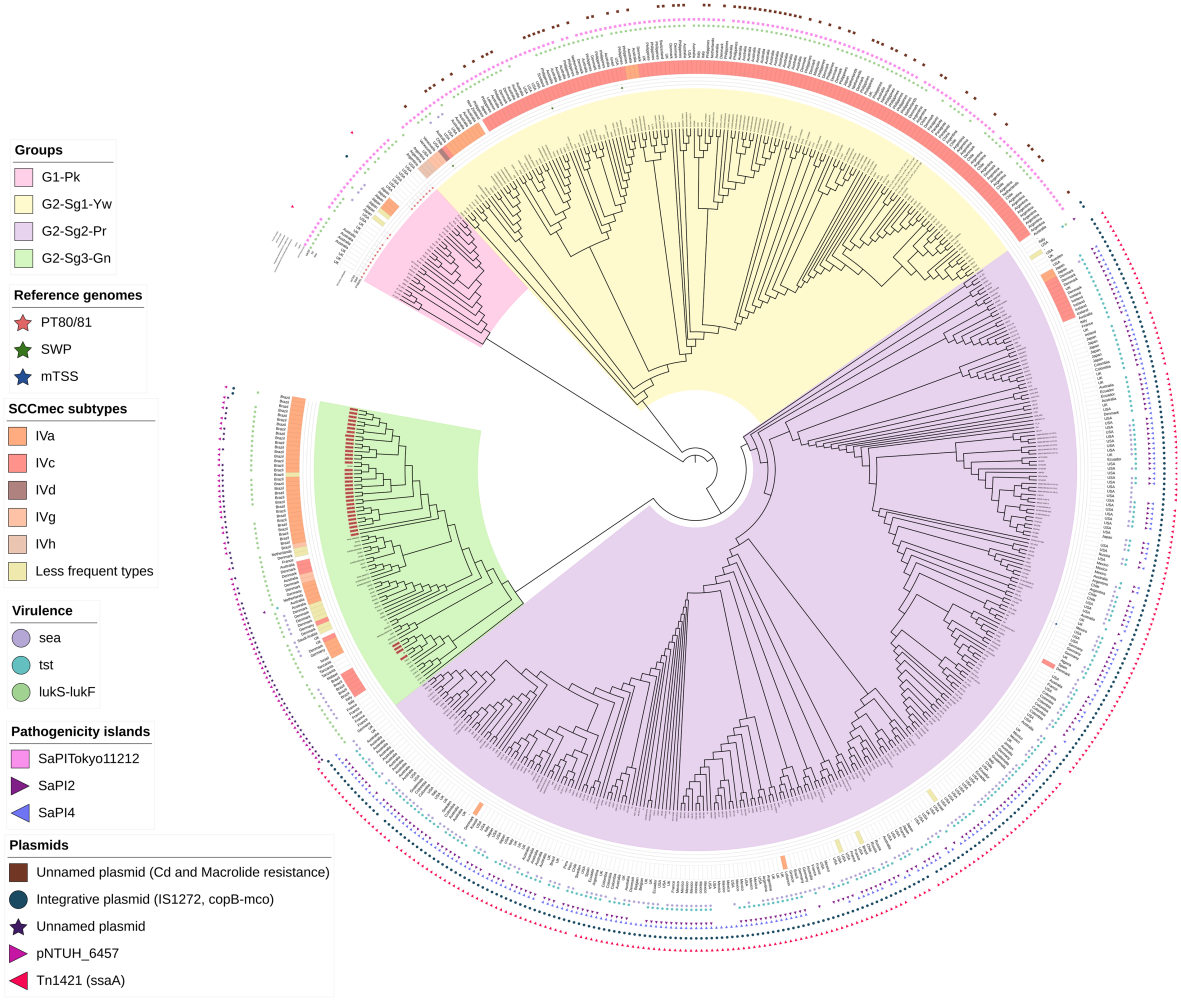
A maximum-likelihood phylogenetic tree was constructed, including 541 genomes classified as ST30 MSSA or MRSA. Phylogenetic groups were defined based on tree topology, with group 1, marked in pink (**G1-Pk**), and group 2 (**G2**), which is further divided into three subgroups: subgroup 1 (Sg1) in yellow (Yw) and designated as **G2-Sg1-Yw**; subgroup 2 (Sg2) in purple (Pr) and named **G2-Sg2-Pr**; and subgroup 3 (Sg3) in green (Gn) and referred to as **G2-Sg3-Gn**. The outer ring of the tree indicates the staphylococcal cassette chromosome *mec* (SCC*mec*) subtypes. Circles display the presence of specific virulence genes: *sea* (encoding enterotoxin A) in purple, *tst* (encoding toxic-shock syndrome toxin-1) in light blue, and *lukS-lukF* (encoding Panton-Valentine leukocidin) in light green. Stars represent genomes of the main *S. aureus* historical groups: Phage-Type 80/81 (PT80/81) in red, Southwest Pacific clone (SWP) in dark green, and menstrual toxic shock syndrome (mTSS) in dark blue. Pink squares represent the presence of Pathogenic Island SaPITokyo11212, purple triangles pointing rich represent SaPI2 and light-purple triangles SaPI4. Brown squares represent the presence of an unnamed plasmid carrying resistance genes for cadmium and macrolide resistance. Blue circles indicate an integrative plasmid containing insertion IS*1277*, the copper-transporting ATPase gene (*copB*), and the multicopper oxidase gene (*mco*). The purple star marks the unnamed plasmid detected in this study, while the light-purple right-pointing triangle represents pNTHU_6457, and the dark-pink left-pointing triangle represents plasmid Tn*1421*. Genome numbers highlighted in red denote those sequenced in this study.

The phylogenetic tree was constructed using 541 ST30 sequences. Genomes were annotated with Prokka v.1.14.6 and the core genome alignments were performed with Roary v.3.13.0 using MAFFT, including only genes present in ≥99% of the genomes, ensuring high-quality alignments. Both Prokka and Roary are accessible at https://github.com/tseemann/prokka and https://github.com/sanger-pathogens/roary, respectively. A maximum likelihood (ML) tree was inferred using RAxML v.8.2.12 accessible at http://github.com/stamatak/standard-RAxML with the GTR-CAT evolutionary substitution model and 100 bootstrap replicates. The GTR-CAT model was chosen for its ability to efficiently handle the large dataset of nearly 550 genomes by accounting for site-specific evolutionary rate variation while maintaining computational feasibility, making it a robust alternative to GTR + *Γ* for large-scale phylogenetic inference (Whelan and Halanych, 2017). The tree was rooted using outgroup genomes from strains clustered in a more basal group, marked in pink in Figure 1. The Interactive Tree of Life (iTOL) v.6.9.1, accessible at http://itol.embl.de/, was employed for tree visualization and editing.

### 2.4 Time-scaled evolutionary analysis

We performed two Bayesian phylogenetic analyses to investigate the evolutionary history and chronology of ST30. The use of assemblies in these analyses is justified by the study’s objective of inferring macro-evolutionary events. Since the analysis focuses on conserved genomic regions, localized errors in assemblies have minimal impact on the accuracy of the chronological estimates. The first included 61 genome representatives of all groups and subgroups. The second analysis focused on genomes from the subgroup clustering Brazilian strains. The genomes included in the BEAST analysis were carefully selected to ensure they represent the major groups, subgroups, and subsets of these subgroups identified in the core genome tree constructed from the 541 genomes. This selection strategy allowed us to capture the most relevant diversity and evolutionary history while ensuring computational feasibility for the time-calibrated analysis. Core genomes were aligned using Roary, and phylogenetic trees were constructed with RAxML. ClonalFrameML v.1.13, accessible at http://github.com/xavierdidelot/ClonalFrameML, filtered polymorphic sites that could influence the Bayesian phylogenetic analysis, which was performed using BEAST v.1.10.4 (Suchard et al., 2018). The resulting trees were visualized and annotated with iTol.

### 2.5 Virulence, resistance, and mobile genetic elements

Virulence contents were analyzed with BLAST command line accessible at http://ncbi.nlm.nih.gov/books/NBK569861, and the Virulence Finder Database (VFDB) accessible at http://mgc.ac.cn/VFs/main.htm, applying a 90% coverage and 95% identity threshold. Due to the paralogs that could lead to annotation errors of *lukSF*-PVL, the reference sequences of these genes were downloaded as contiguous sequences (strain 13420; Acc: NZ_CP021141.1, nucleotide (nt) position 1,518,664–1,520,582). Genomic islands (GIs) *v*Saα, *v*Saβ, *v*Saγ, SaPI1, SaPI3, SaPI4, and IEC types A–F were initially identified in reference genomes using specific biomarkers (detailed in Supplementary File S1). The open reading frames (ORFs) of each GI and SaPI were extracted using the Map to Reference tool in Geneious Prime Software. These reference sequences were then used to analyze the 541 genomes via the BLAST command line, applying thresholds of 95% identity and 90% coverage. PVL haplotype sequences were obtained from the study by O’Hara et al. (2008). These sequences were extracted from the 541 genomes and used as queries in BLAST command-line searches, with thresholds set at 90% coverage and 100% identity threshold. Genomes that could not be precisely typed with 100% identity were assigned to the haplotypes showing the closest match. Acquired antimicrobial resistance genes were identified using the CARD database via ABRicate, accessible at http://github.com/tseemann/abricate, with a minimum identity of 90% threshold.

### 2.6 Pangenomic analysis

An open-reading frame (ORF)-based pangenomic binary (1, 0) matrix was used to assess ORF differences and the presence/absence of DNA blocks, such as mobile genetic elements (MGEs). The matrix was constructed as previously described (Esteves et al., 2023), with pangenome groups formed by each group or subgroup. The reference strains used were as follows: NCTC11561 (Acc.: GCA_900458105.1) for the clade grouping PT80/81, Sa-122 (Acc.: GCA_028389215.1) for subgroup 1 of the contemporary group, MN8 (Acc.: GCA_022163365.1) for subgroup 2 of the contemporary group, and CR14-021 (Acc.: GCA_021012415.1) for subgroup 3 of the contemporary group. To construct the matrix, BLAST command-line searches were performed using 90% coverage and 100% identity. A Student’s *t*-test was conducted, and ORF sequences with overall significance above the cutoff (−log_10_ *p*-value ≥10) were localized in the reference genomes using the Map to Reference tool in Geneious Prime. A detailed description of the Material and Methods section is available in Supplementary File S1.

## 3 Results and discussion

### 3.1 Phylogenetic and genomic analyses

A phylogenetic tree was constructed using 541 ST30 genomes from MSSA/MRSA strains isolated from 1958 to 2022. The *mecA* gene was detected in 44.7% (*n* = 242/541) of the genomes (Table 1), which is consistent with the global MRSA rate in some countries such as the USA (Kourtis et al., 2019). However, we cannot rule out the possibility of bias in the selected genome sequences. The structure of this tree indicates that the evolutionary process led to the diversification of the original ST30 ancestor into two distinct groups: (*i*) group 1 (**G1**), depicted in pink (Pk), and referred to as **G1-Pk**; and (*ii*) group 2 (**G2**), which comprises: subgroup 1 (Sg1), colored yellow (**G2-Sg1-Yw**); subgroup 2 (Sg2), colored purple (**G2-Sg2-Pr**); and subgroup 3 (Sg3), colored green (**G2-Sg3-Gn**) (Figure 1; Supplementary Figure S1).

**Table 1:** Frequency of the predominant *spa* types and SCC*mec* types in the genomes grouped in each group/subgroup described in this study.

	Number of *spa* typable/total genomes (%)	*spa* types (%)^a^	Number of MRSA/total genomes (%)	Number of SCC*mec* type/total MRSA (%)^a^
**G1-Pk**	24/28 (85.7)	t1209: 14/28 (50.0) t021: 4/28 (14.3) t233: 4/28 (14.3)	5/28 (17.9)	IVd: 3/5 (60.0) Ia: 2/5 (40.0)
**G2-SG1-Yw**	133/155 (85.8)	t019: 108/155 (69.7)	154/155 (99.3)	IVc: 135/154 (87.7)
**G2-SG2-Pr**	255/281 (90.7)	t012: 65/281 (23.1) t21: 43/281 (15.3) t338: 38/281 (13.5)	19/281 (6.8)	IVc: 11/19 (57.9) IVa: 4/19 (21.0)
**G2-SG3-Gn**	60/77 (77.9)	t02: 19/77 (24.7) t318 9/77 (11.7)	64/77 (83.1)	IVa: 41/64 (64.1) IVc: 11/64 (17.2)

a Only the predominant types and allelic patterns were reported for the groups and subgroups of the ML phylogenetic tree.

**G1-Pk, group 1 in pink; G2-Sg1-Yw, group 2, subgroup 1, in yellow; G2-Sg2-Pr, group 2, subgroup 2, in purple; G2-Sg3-Gn, group 2, subgroup 3, in green; t, spa type; SCCmec, staphylococcal cassette chromosome mec; MRSA, methicillin-resistant Staphylococcus aureus.**

**Figure 2:**
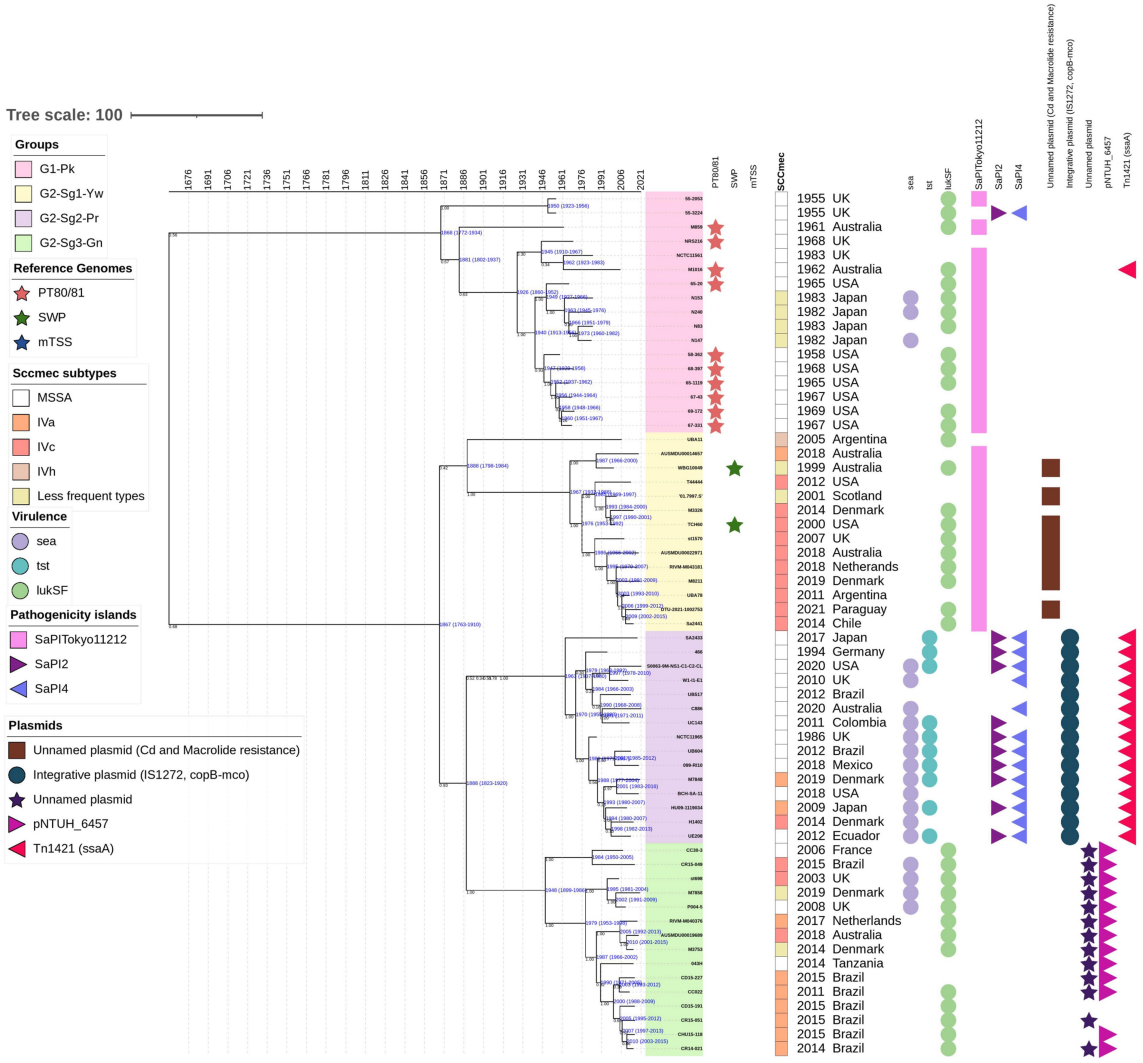
The time-calibrated Bayesian phylogenetic tree was constructed with 61 representative genomes from all groups and subgroups included in the phylogenetic analysis performed on the 541 genomes. The maximum clade credibility tree was estimated using an uncorrelated log-normal relaxed clock model, starting with a random tree and employing a coalescent constant tree prior. The colors of the isolate numbers correspond to the same colors used in the text and depicted in the ML phylogenetic tree. Values in black indicate the posterior probability for each node, while values in blue represent the age in years along with the 95% highest posterior density interval (HPDI) for each group. Reference genomes are indicated in colored stars: phage-type 80/81 in red, Southwest Pacific (SWP) clone in dark green, and menstrual toxic shock syndrome (mTSS) in dark blue. Rectangles denote the staphylococcal cassette chromosome *mec* (SCC*mec*) subtypes, represented in different colors. Circles indicate the presence of specific genes: *sea* (encoding enterotoxin A), *tst* (encoding toxic-shock syndrome toxin-1), and *lukS-lukF* (encoding Panton-Valentine leukocidin). Pathogenicity islands are represented as follows: SaPITokyo*11212* in pink squares, SaPI2 in purple triangles, and SaPI4 in blue triangles. Plasmids are also depicted: an unnamed plasmid carrying genes for cadmium and macrolide resistance in brown squares; an integrative plasmid with two copies of insertion sequence IS*1272*, copper-transporting ATPase (*copB*), and multicopper oxidase (*mco*) in blue circles; an unnamed plasmid in purple stars; the pNTHU_6457 plasmid in light-purple right-pointing triangles; and a plasmid carrying a Tn*1421* element with a second copy of *ssaA* in dark-pink left-pointing triangles.

According to the chronology model applied in this study, the evolutionary split that gave rise to PT80/81 strains, which are grouped in **G1-Pk**, is estimated to have occurred around 1868 [95% highest posterior density interval (HPDI) 1772–1934]. This split also marks the origin of **G2,** estimated to have emerged in 1867 (95% HPDI 1763–1910), which encompasses strains related to SWP, grouped in **G2-Sg1-Yw** (Figure 2). These two groups seem to have evolved concurrently after their divergence from a common ancestor. By examining eight genomes of PT80/81 and SWP, DeLeo et al. (2011) also suggested a divergence between these epidemic *S. aureus* lineages. Similarly, McAdam et al. (2012) reached the same conclusion in their phylogenetic analysis of 87 CC30, despite the majority being ST36 (CC30)-SCC*mec*II (*n* = 60), with only 17 genomes related to PT80/81 and just 4 SWP.

In this study, which includes a significantly larger collection of recent ST30 genomes from various regions, we provide a more detailed view of the dynamics of shifts across the phylogeny. Additionally, the relationship between PT80/81, SWP, and more recent ST30 strains is explored in greater depth. Our phylogenetic tree showed that the majority of contemporary ST30 isolates, encompassing both MRSA and MSSA strains, clustered within **G2** subgroups. Although PT80/81 and SWP are part of the same ST30 lineage, their diversification possibly led to an increase in virulence and host adaptability among the more common and globally disseminated contemporary ST30 strains found in the **G2** (Figure 1; Supplementary Figure S1).

Notably, the tree structure indicates a continuous intercontinental flow of **G2** strains, marked by an intense worldwide spread. This pattern may be attributed to the high prevalence of ST30 infections among previously healthy non-hospitalized individuals, which could facilitate the widespread distribution of these strains over long distances (Ribeiro et al., 2005; Ko et al., 2013; Macal et al., 2014; Barcudi et al., 2024; Ferreira et al., 2021).

### 3.2 The historical group G1-Pk

This group includes the pandemic strains of PT80/81—depicted with red stars in Figure 1 and Supplementary Figure S1— along with related strains (*n* = 28). Using a Bayesian time-calibrated model (Figure 2), we traced the expansion of PT80/81 strains back to their common ancestor with an estimated date of 1926 (95% HPDI 1860–1952). It has been said that PT80/81 MSSA emerged around the 1950s, rapidly causing a 10-year pandemic, that resulted in severe HA and CA infections in several countries including Australia, Great Britain, Canada, and the United States (Rountree and Beard, 1958; Bynoe et al., 1956; Robinson et al., 2005; DeLeo et al., 2011). Despite this, Blair and Carr (1960) reported that PT80/81 was a significant cause of infections prior to its rise to prominence; they examined 94 *S. aureus* strains isolated from 1927 to 1947, revealing that 43 strains were PT80/81, with the earliest strain isolated in 1927. These studies support the time-calibrated model proposed here, which suggests that the expansion began around 1926 (Figure 2).

Remarkably, MRSA strains from Japan (N296, N240, N153, N147, and N83), collected between 1982 and 1983 (Matsumoto et al., 1984), are closely related to PT80/81. Additionally, phylogenetic studies by Zuo et al. (2021) further support the clustering of Japanese MRSA with PT80/81 strains (Figure 1; Supplementary Figure S1). The MRSA Japanese genomes harbored SCC*mec*IVd (*n* = 3) or SCC*mec*Ia (*n* = 2). Although the **G1-Pk** group was based on a limited number of strains, these findings suggest at least two introductions of SCC*mec* through horizontal gene transfer (Figure 1; Supplementary Figure S1). These strains shared a common ancestor in 1963 (95% HPDI 1945–1976), shortly after methicillin commercialization (Figure 2). Indeed, the first MRSA isolates were described by Jevons (1961). Strains in **G1-Pk** may also produce PVL (71.4%; *n* = 20/28), predominantly classified as the H2a haplotype (55%; *n* = 11/20), primarily carried by the PVL phage φPVL108 (Table 2), which has been previously linked to PT80/81 (Chen et al., 2013).

**Table 2:** PVL phages (**φ**PVL) and PVL haplotypes detected in each group and subgroups of the ML phylogenetic tree.

Group	PVL-positive/total genomes (%)	PVL phage (%)	Haplotype/total PVL (%)
**G1-Pk**	20/28 (71.4)	φPVL108: 18/20 (90.0) φ5967PVL: 1/20 (5.0)NT: 1/20 (5.0)	H2a: 11/20 (55.0) NT (45.0)
**G2-Sg1-Yw**	119/155 (76.8)	φPVLCN125: 87/119 (73.1) φPVL108: 20/119 (16.8) NT: 12/119 (10.1)	H2a 117/119 (98.3) H1a: 2/119 (1.7)
**G2-Sg2-Pr**	1/281 (0.4)	φ5967PVL: 1/1 (100.0)	H1a: 1/1 (100.0)
**G2-Sg3-Gn**	66/77 (85.7)	φ5967PVL: 35/66 (53.0) φPVL108: 3/66 (4.5) φSa2USA: 1/66 (1.5) NT: 27/66 (40.9)	H2b: 46/66 (69.7) H1a: 20/66 (30.3)

The number in parentheses represents the percentage of PVL phage and PVL haplotype found in PVL-positive isolates for each group and subgroups of ML phylogenetic tree. **G1-Pk**, group 1, in pink; **G2-Sg1-Yw**, group 2, subgroup 1, in yellow; **G2-Sg2-Pr**, group 2, subgroup 2, in purple; **G2-Sg3-Gn**, group 2, subgroup 3, in green; PVL, Panton-Valentine leukocidin; NT, not typeable.

A proposed set of gene alleles for CC30 classification includes *agrC*, *hla* (DeLeo et al., 2011), and *isdH* (McGavin et al., 2012). DeLeo et al. (2011) studied eight CC30 genomes of PT80/81, SWP, and contemporary CC30 hospital strains including ST36-MRSA and ST30-MSSA strains, identifying specific nucleotide substitutions in *agrC* and *hla* associated only with the so-called contemporary CC30. The authors suggested that mutations in these genes in contemporary strains reduced virulence, confining this pathogen to hospital settings, while wild-type (WT) alleles were essential for the global prevalence of PT80/81 until the 1960s (DeLeo et al., 2011; Gasch et al., 2012). McGavin et al. (2012) also found that these mutations correlated with a specific SNP in *isdH*. However, MRSA ST30 strains were not included in their studies. In our study, we found WT alleles in contemporary MRSA ST30 hospital isolates from various phylogenetic groups including **G2-Sg1-Yw** and **G2-Sg3-Gn**, while the mutant allele profile was exclusively present in MSSA strains of the **G2-Sg2-Pr**, which comprised most of the sequenced genomes (Table 3; Figure 1; Supplementary Figure S1). Corroborating our findings, strain MN8, identified by DeLeo et al. (2011) as having mutant alleles, clustered within **G2-Sg2-Pr**. McAdam et al. (2012) reported that ST36(CC30)-SCC*mec*II strains also exhibited this mutated profile; however, ST36 strains were not included in our study.

**Table 3:** Significant genomic events that occurred during the evolution of ST30 strains.

Genetic elements	G1-Pk	G2-Sg1-Yw	G2-Sg2-Pr	G2-Sg3-Gn
φPVL108	+	−	−	−
SaPITokyo11212-like	+	+	−	−
SCC*mec*IV	−	+	−^a^	+
Lipoprotein-like complete cluster in *v*Saα	−	+^b^	+^b^	+^b^
Unnamed plasmid (Cd and Macrolide resistance)	−	+	−	−
ORFs near ESAT cluster	−	+^c^	+^c^	+^c^
Tn*1421* element carrying a second copy of *ssaA*^d^	−	−	+	−
Integrative plasmid carrying two copies of IS*1272*, and *copB-mco*	−	−	+	−
SaPI2 carrying *tst*	−	−	+	−
SaPI4	−	−	+	−
SaPI-14-021	−	−	−	+
pNTUH_6457	−	−	−	+
Toxin-antitoxin (*toxM*)	+	+	−	+
*agrC*/*hla*/*isdH* mutated alelle^e^	−	−	+	−
Unnamed plasmid	−	−	−	+
SraP-A^f^	+	−	−	−
SraP-B	−	+	−	−
SraP-C	−	−	+	−
SraP-D	−	−	−	+

a Most strains (87.6%) are MSSA.

b Although some *lpl* genes can be absent, the *lpl* cluster is more complete in **G2** subgroups compared to that of **G1-Pk**.

c While some differences were observed between **G2-Sg2-Pr** and the other subgroups, the identity among **G2** genomes is much higher than that observed among those in **G1-Pk**.

+, presence; −, absence; **G1-Pk**, group 1 pink; **G2-Sg1-Yw**, group 2, subgroup 1, yellow; **G2-Sg2-Pr**, group 2, subgroup 2, purple; **G2-Sg3-Gn**, group 2, subgroup 3, green.

d Tn*1421* is a newly identified conjugative transposon carrying a homolog of the *ssaA* gene, found exclusively in **G2-Sg2-Pr** strains.

e *agrC, encodes the accessory gene regulator C; hla, encodes the hemolysin A; isdH, encodes the iron-regulated surface determinant protein H; WT, wild-type.*

f SraP-A to D represents the various SraP polymorphism of ST30 strains.

**Figure 3:**
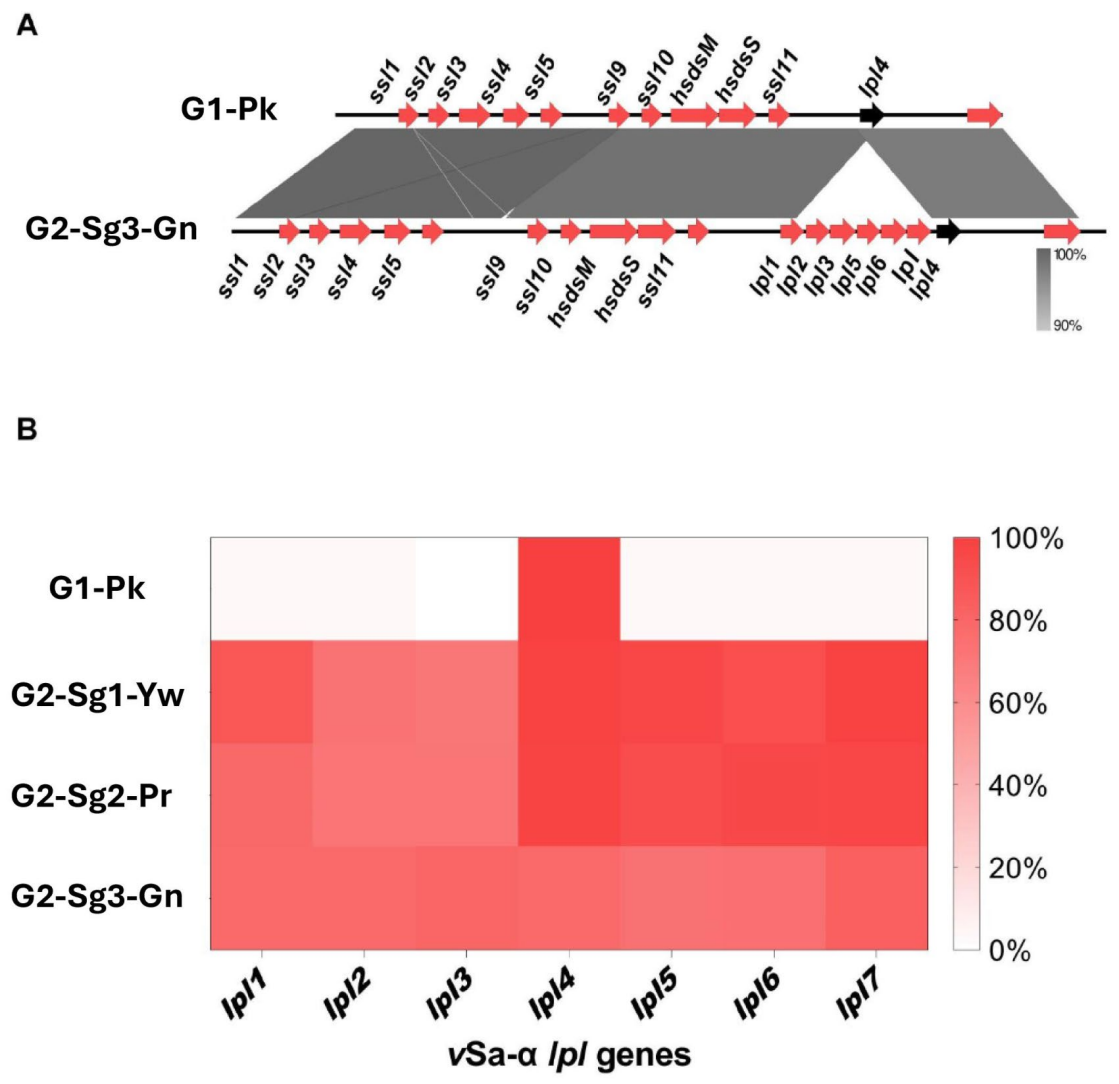
Figures indicating the absence of *lpl* genes encoding for lipoprotein-like proteins in the *ν*Saα genomic island. **(A)** Genomic alignment between a *ν*Saα genomic island from representative genomes from **G1-Pk** and from **G2-Sg3-Gn**. Gray shading indicates the identity percentage between sequences, and red arrows indicate the ORFs carried by *ν*Saα island. The black arrow highlights the *lpl4* gene, which is the only *lpl* gene common to both groups. **(B)** Heatmap representing the frequency of genomes from each group or subgroups that present the *lpl* genes carried in *ν*Saα, evidencing the presence of *lpl4* and the absence of other *lpl* genes in **G1-Pk**. **G1-Pk**: group 1 pink; **G2-Sg1-Yw**: group 2, subgroup 1, yellow; **G2-Sg2-Pr**: group 2, subgroup 2, purple; **G2-Sg3-Gn**: group 2, subgroup 3, green; *lpl*: lipoprotein-like encoding gene; *ssl*: staphylococcal superantigen-like encoding gene.

Another important finding was the absence of *lpl* genes (except *lpl4*) in the genomic island (GI) *ν*Saα among strains from **G1-Pk**, in contrast to the more frequent presence of the complete *lpl* cluster for the subgroups of **G2** (Figures 3A,B). This cluster is involved in the synthesis and modification of lipoproteins, contributing to bacterial virulence, cell invasion, and immune evasion (Nguyen et al., 2016). Previous studies with *lpl* knockout in the USA300 background demonstrated the role of *lpl* genes in cell invasion and immune response and showed that Lpls act as cyclemodulins leading to the G2/M phase transition delay in HeLa cells (Nguyen et al., 2016). Although this hypothesis was not tested for ST30, given the importance of *lpl* genes for bacterial virulence and host adaptation, it is reasonable to suppose that the absence of most *lpl* genes in PT80/81-related strains may have played a role in limiting the pandemic potential of these strains and restricting their global spread.

**Figure 4:**
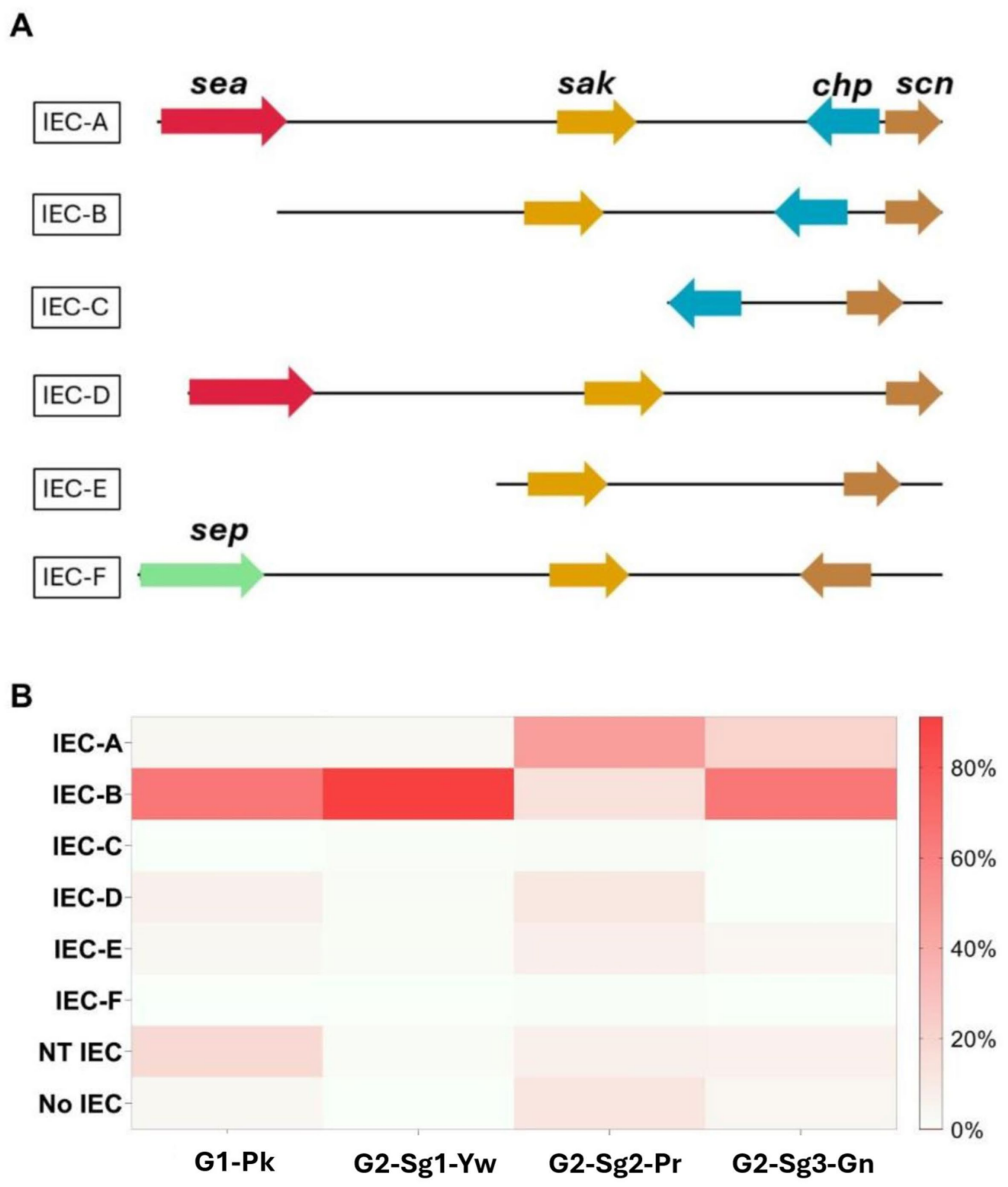
Representation of the distribution and genomic context of the different types of immune evasion cluster (IEC) in ST30 genomes included in this study. **(A)** Genomic representation of the genes grouped in the IEC cluster, based on the presence of *sea* (encoding staphylococcal enterotoxin A), *sak* (encoding staphylokinase), *chp* (encoding chemotaxis inhibitory protein), *scn* (encoding staphylococcal complement inhibitor), and *sep* (encoding staphylococcal enterotoxin P) genes. **(B)** Heatmap represents the frequencies of each type of IEC in the phylogenetic groups and subgroups. NT, non-typeable; No, no IEC was identified; **G1-Pk**, group 1 pink; **G2-Sg1-Yw**, group 2, subgroup 1, yellow; **G2-Sg2-Pr**, group 2, subgroup 2, purple; **G2-Sg3-Gn**, group 2, subgroup 3, green.

Additional GI, the *v*Saβ type-III, featuring a complete enterotoxin gene cluster (*egc*) but an incomplete *spl* cluster (Kläui et al., 2019), was found in **G1-Pk** and most ST30 genomes. The immune evasion cluster (IEC) type-B, which lacks *sea* and *sep*, predominated in **G1-Pk** and most of the ST30 genomes analyzed (Figure 4). IEC is carried by β-hemolysin-converting bacteriophages (Sa3int phages) and harbors the gene *scn* (encoding staphylococcal complement inhibitor) in different combinations with *sak*, *chp*, *sea*, and *sep*, which encodes staphylokinase, chemotaxis inhibitory protein (CHIPS) and staphylococcal enterotoxin A and P, respectively, playing a pivotal role in inhibiting opsonic phagocytosis (Rohmer and Wolz, 2021).

**Figure 5:**
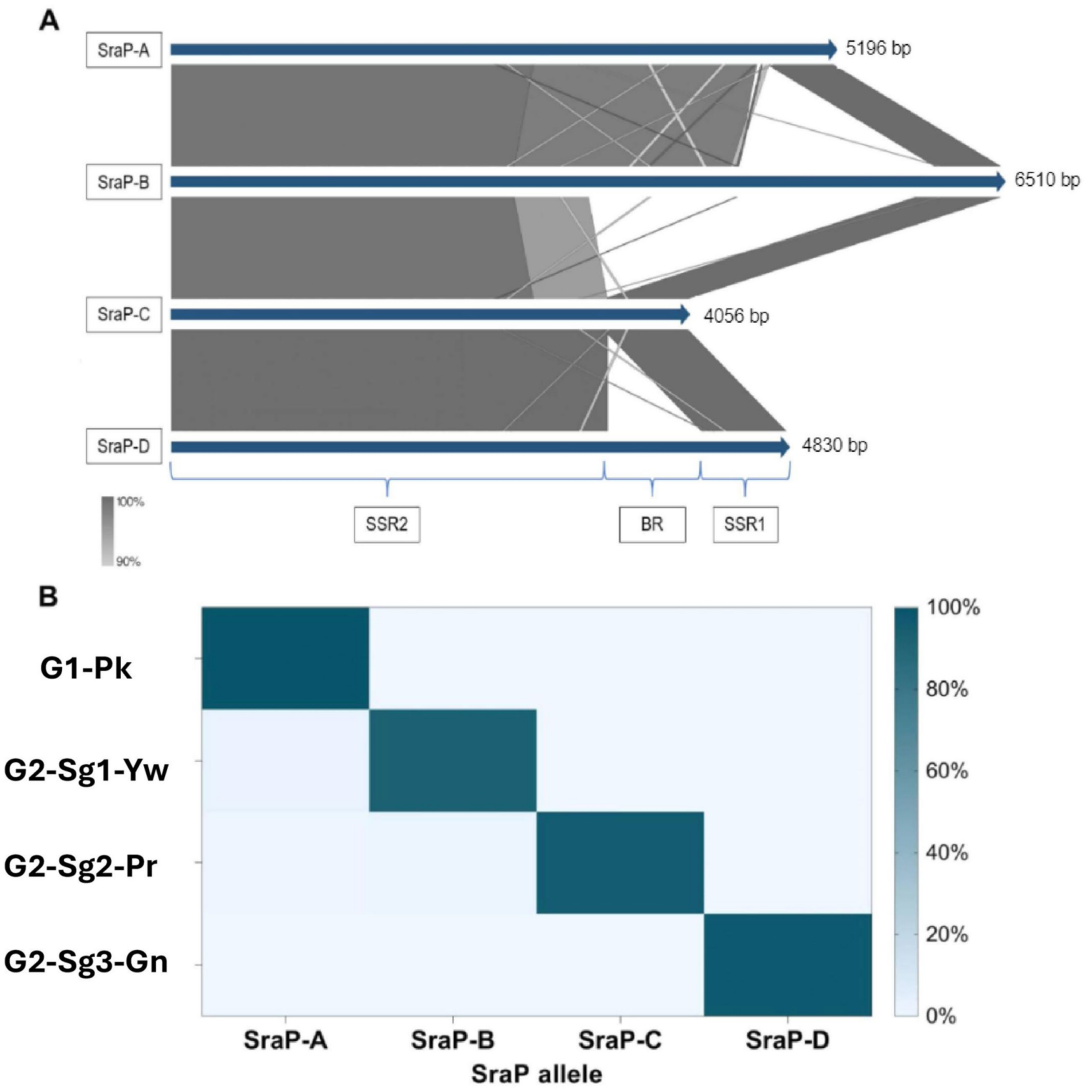
Representation of the distribution and genetic context of different types of *sraP* gene found in ST30 genomes. **(A)** Alignment of the different *sraP* types with their respective lengths measured in base pairs (bp) for each group, based on the percentage identity of each region of the *sraP* gene; SRR1: conserved region 1; BR: variable region coding for the ligand region of the protein and SRR2: conserved region 2. **(B)** Heatmap representing the frequencies of each *sraP* type in the phylogenetic groups and subgroups. **G1-Pk**, group 1 pink; **G2-Sg1-Yw**, group 2, subgroup 1, yellow; **G2-Sg2-Pr**, group 2, subgroup 2, purple; **G2-Sg3-Gn**, group 2, subgroup 3, green.

Remarkably, the gene *sraP*, encoding a serine-rich adhesin for binding platelets (SraP), exhibited a cladistic polymorphism in ST30 genomes. In this study, we classified *sraP* alleles based on the apomorphy of the variable region (BR), ranging from 784pb and 3238pb, and flanked by the conserved regions SSR1 and SSR2 (Yang et al., 2014). The proposed classification included *sraP*-A in **G1-Pk**; *sraP*-B for **G2-Sg1-Yw**; *sraP*-C for **G2-Sg2-Pr**; and *sraP*-D for **G2-Sg3-Gn** (Figure 5). This protein is highly conserved in *S. aureus* and has been linked to infective endocarditis (Siboo et al., 2005), although the function of this polymorphic region in platelet adhesion remains unclear. The apomorphy of SraP proteins, along with the tree structure (Figure 1), supports the hypothesis that the evolution of ST30 led to the emergence of few clonal groups capable of spreading to distant geographic regions through intense multidirectional flow.

ST30 strains are generally susceptible to non-β-lactam antibiotics (Macedo-Viñas et al., 2014; Viana et al., 2021); although the reason for this remains unclear. The *tet*(38) gene, associated with resistance to tetracycline, was found in the majority of ST30 genomes (99.1%; *n* = 536/541), though previous studies showed only 2 out of 109 strains phenotypically resistant to this drug (Viana et al., 2021). Tet(38) is a member of the major facilitator superfamily (MFS) of efflux membrane, homologous to TetK/L, that plays an important role in *S. aureus* invasion and survival in epithelial cells, through possible interactions with CD36 (Truong-Bolduc et al., 2017). Thus, it may contribute to the virulence attributable to ST30 strains. Resistance genes *ermA* and *ermB* (erythromycin and macrolide, lincosamide and streptogramin B), *tetK* (tetracycline resistance), and *dfrG* (trimethoprim resistance) were found at low frequency in the 541 ST30 genomes analyzed, although few clusters showed higher percentages of 46.4% (*n* = 13/28; **G1-Pk**), 14.3% (*n* = 04/28; **G1-Pk**), 21.4% (*n* = 06/28; **G1-Pk**), and 33.8% (*n* = 26/77; **G2-Sg3-Gn**); respectively (Supplementary Table S2).

### 3.3 Dissecting the group G2

This group, comprising the subgroups **G2-Sg1-Yw** with the SWP strains, **G2-Sg2-Pr**, and **G2-Sb3-Gn**, likely originated from the specialization of a common ancestor. The subgroup **G2-Sg1-Yw**, clusters SWP strains along with South American strains from Argentina. **G2-Sg2-Pr** primarily clusters MSSA strains that carry the *tst* gene for toxic shock syndrome toxin-1 (TSST-1) but lack the PVL genes. **G2-Sg3-Gn** includes most of the Brazilian strains from the current study. Although Brazil and Argentina share a border, strains from these countries are categorized into distinct phylogenetic subgroups (Figure 1; Supplementary Figure S1).

#### 3.3.1 The evolution of subgroup G2-Sg1-Yw

The 155 strains, representing 28.7% of the 541 genomes analyzed, grouped in **G2-Sg1-Yw**, were isolated between 2001 and 2020. Most strains in this group were from Australia (23.9%; *n* = 37/155), the Philippines (19.4%; *n* = 30/155), and Argentina (16.1%; *n* = 25/155). Among the strains with reported isolation sites (58.1%; *n* = 90/155), the majority (63.3%; *n* = 57/90) were recovered from skin/soft tissue infections (SSTI), and 15.6% (*n* = 14/90) from blood. Nearly all strains in this subgroup are MRSA (99.4%; *n* = 154/155), with SCC*mec* type IVc predominating (87.7%; *n* = 135/154). Other SCC*mec* subtypes were detected at lower frequencies, suggesting multiple gain and loss events of SCC*mec* into this subgroup, similar to what was observed in **G1-Pk**. Among the typable genomes, the predominant *spa* type was t019 (Table 1). This subgroup is characterized by a cluster of strains from SWP (e.g., strain WBG10049), marked with green stars in Figure 1 and Supplementary Figure S1. SWP and Argentinian strains shared a common ancestor in 1967 (95% HPDI 1932–1988) (Figure 2), consistent with McAdam et al. (2012) findings that SWP strains shared a common ancestor in 1967 (95% HPDI 1952–1984).

Key evolutionary events in the specialization of **G1-Pk** and **G2** include changes in GIs and the acquisition, by the strains in **G2-Sg1-Yw**, of an unnamed plasmid (Acc: CP053637.1) that carries the *mphC* gene associated with macrolide resistance and the *cadD* gene for cadmium resistance. The loss of a novel *S. aureus* pathogenicity islands (SaPI), SaPITokyo11212-like, occurred during the split between **G2-Sg1-Yw** and the other **G2** subgroups around 1888 (95% HPDI 1823–1920); thus, it is present only in genomes **G1-Pk** and **G2-Sg1-Yw** (Table 3).

**Figure 6:**
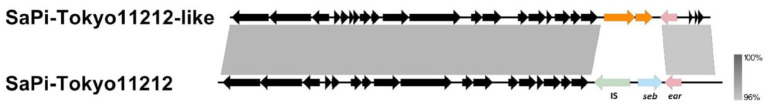
Alignment between SaPITokyo11212 and SaPITokyo11212-like elements found in genomes grouped in **G1-Pk** (group 1, pink) and **G2-Sg1-Yw** (group 2, subgroup 1, yellow). The figure shows the absence of the transposase (IS, light green) and the *seb* gene (light blue) in the SaPI found in **G1-Pk** and **G2-Sg1-Yw** and indicates the presence of the *ear* gene in both SaPIs. Gray shading indicates the percentage of nucleotide identity between the two sequences.

The SaPIs of CC30 strains were previously detailed by McGavin et al. (2012), who identified four main pathogenicity islands (SaPI-1 to 4). In fact, the SaPI1 identified by those authors shows higher nucleotide identity and coverage with SaPITokyo11212, as described by Suzuki et al. (2015). SaPITokyo11212 contains the *ear* and *seb* genes, where *seb* encodes enterotoxin B, which causes food poisoning, and *ear* encodes an exoprotein predicted to act as a superantigen. However, the deletion of the *ear* gene did not significantly impact a mouse model (Singh et al., 2017). The novel SaPITokyo11212-like lost the *seb* gene and the adjacent transposase IS*256* (Figure 6). Therefore, the absence of virulence-associated genes may explain why this island was not conserved in other **G2** subgroups. SaPI2, which carries the *tst* gene, was present in most genomes within **G2-Sg2-Pr**. In all *tst*-positive strains, the *tst* gene was harbored by SaPI2-like elements similar to those found in MN8 ST30 strain, which carry *tst* as the sole virulence gene (McGavin et al., 2012). SaPI3 was absent from all ST30 genomes. SaPI4, which also lacks virulence genes, was identified exclusively in **G2-Sg2-Pr**, a group containing MN8-related strains (Figure 6).

**Figure 7:**
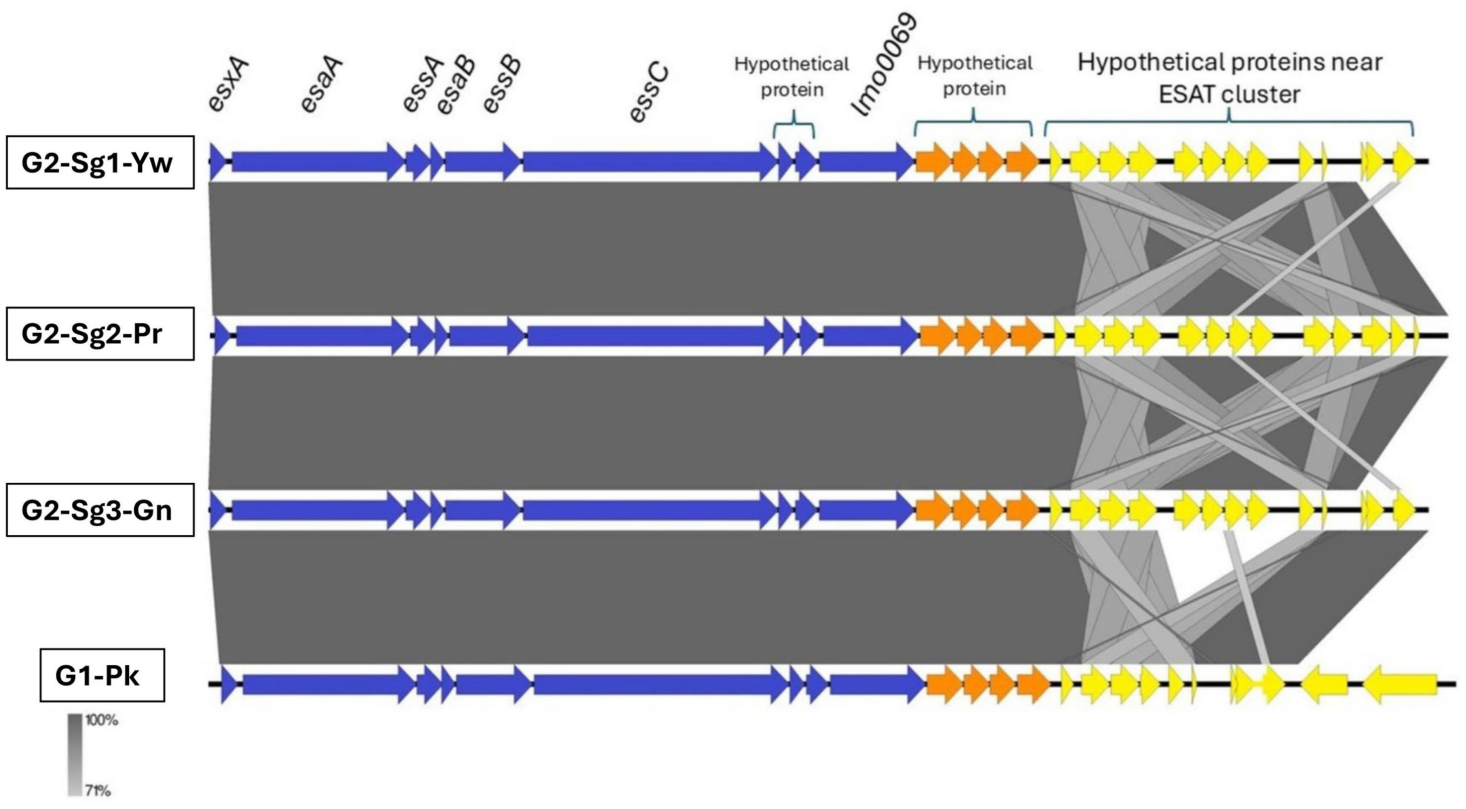
Genomic comparison of representative sequences of the ESAT-6 secretion system cluster and downstream coding sequences. Blue arrows represent the ESAT genes; orange arrows represent hypothetical protein-coding sequences; and yellow arrows indicate the genes coding for proteins near the ESAT cluster. *esxA*, encodes type VII secretion system extracellular protein A; *esaA*, encodes type VII secretion system accessory factor EsaA; *essA*, encodes ESAT-6 secretion machinery protein EssA; *esaB*, encodes type VII secretion system component EsaB; *essB*, encodes type VII secretion system protein EssB; *essC*, encodes type VII secretion system protein EssC; *lmo0069*, encodes putative Lmo0069-like protein, LXG domain-containing protein *Staphylococcus aureus* (strain MRSA252); **G1-Pk**, group 1 pink; **G2-Sg1-Yw**, group 2, subgroup 1, yellow; **G2-Sg2-Pr**, group 2, subgroup 2, purple; **G2-Sg3-Gn**, group 2, subgroup 3, green.

A region with hypothetical open reading frames (ORFs) named “proteins near ESAT” showed greater conservation among genomes of **G2** subgroups (particularly **G2-Sg1-Yw** and **G2-Sg3-Gn**) compared to **G1-Pk** (Figure 7). In *S. aureus*, the ESAT-6-like secretion system (ESS), comprising 12 or more genes, facilitates type VII secretion (TVIIS) across the bacterial cell wall. ESS is crucial for secreting virulence factors during infections, influencing host immune responses by altering cytokine production (Anderson et al., 2016). However, the specific role of proteins located near ESAT region remains to be determined.

The *lukSF* genes encoding PVL in the yellow subgroup (76.8%; *n* = 119/155) primarily belong to the H2a haplotype (98.3%; *n* = 117/119), like **G1-Pk**. However, unlike **G1-Pk** strains, where the main phage carrying the PVL haplotype H2a was φPVL108, most strains in **G2-Sg1-Yw** carry PVL by φPVL-CN125 (73.1%; *n* = 87/119), with only a few associated with φPVL108 (16.8%; *n* = 20/119) (Table 2). Previous studies have reported similar phenomena of different phages carrying the same PVL haplotype (Di Gregorio et al., 2021; Coombs et al., 2020). Indeed, Di Gregorio et al. (2021) have also identified φPVL-CN125 as being associated with the prominent ST30 Argentinian cluster ARG-4, which encompassed genomes that clustered within **G2-Sg1-Yw** in our phylogenetic tree. Like **G1-Pk**, this subgroup carried mostly IEC type-B (93.5%; *n* = 145/155) (Figure 4). Strains of this subgroup carry *v*Saα with a more complete *lpl* locus (Figure 3) and *v*Saβ type-III. Additionally, 92.9% (*n* = 144/155) of all genomes in this subgroup were classified as *sraP-B*, featuring a variable region of 3,238 bp, as illustrated in Figure 5.

#### 3.3.2 The evolution of TSST-1 producers in G2-Sg2-Pr

Most genomes in our phylogenetic tree clustered within this subgroup (51.9%; *n* = 281/541). Among the strains with reported isolation region, most were from the USA (30.6%; *n* = 83/271), the UK (11.8%; *n* = 32/271), and Australia (11.8%; *n* = 32/271), with samples collected from 1980 to 2022. Notably over 80% (*n* = 232/281) of the strains were isolated between 2010 and 2020. Most of the strains were MSSA (93.2%; *n* = 262/281) and did not possess *lukSF*, except for one strain (SAO23, Acc.: GCA_028363555.1), which carried the PVL haplotype H1a (Table 2) associated with φ5967PVL. The subgroup exhibited considerable variability in *spa* types, with the most common being t012 (23.1%; *n* = 65/281), t021 (15.3%; *n* = 43/281), and t338 (13.5%; *n* = 38/281) (Table 1). Among the 19 MRSA strains in this subgroup, various SCC*mec* subtypes were identified: SCC*mec*IVc (*n* = 11), SCC*mec*IVa (*n* = 4), and SCC*mec*IIa/IIb (*n* = 2), with two strains being untypable. The SraP-C polymorphism serves as a cladistic biomarker for this subgroup with a variable region of 784 bp (Figure 5).

These strains possess *v*Sa*α* with a more complete *lpl* locus (Figure 3B) and *v*Saβ type-III, sharing most virulence-associated factors with other ST30 genomes. However, their genomes have expanded through significant acquisition such as SaPI2, which encodes the *tst* gene for TSST-1, present in 79.4% (*n* = 223/281) of the genomes analyzed. This is an important evolutionary trait, as all *tst*-positive strains, except one, are concentrated in this subgroup.

Studies estimate the annual incidence of toxic shock syndrome (TSS) to be between 0.03 and 0.07 cases per 100,000 population (Adams et al., 2017). Historically, ST30-MSSA strains have been associated with TSS, exemplified by the historical strain MN8 (depicted with a blue star in Figure 1 and Supplementary Figure S1), which is also clustered within this subgroup. Isolated in 1980 during a pandemic of menstrual toxic shock syndrome (mTSS) (Reingold, 1982), MN8 was part of 867 reported cases in the USA, with 492 cases in 1981. Eighty-eight cases resulted in death, leading to a case-fatality ratio of 5.6% among those with known outcomes, including 15 cases in 1981 (case-fatality ratio of 3.3%) (Cibulka, 1983). The incidence of mTSS significantly decreased after the withdrawal of highly absorbent tampons from the market. More recently, the reported incidence of mTSS ranges from 0.03 to 0.50 cases per 100,000 people, with an overall mortality rate of approximately 8% (Berger et al., 2019).

SaPI2 is estimated to have been acquired around 1963 (95% HPDI: 1937–1980) by **G2-Sg2-Pr** strains, coinciding with the absence of the *lukSF* gene in the analyzed genomes. The first non-menstrual TSS was described in the USA by Todd et al. (1978), though they speculated that it may have been observed earlier, corroborating the chronology presented in our study. SaPI2 appears to have entered exclusively into **G2-Sg2-Pr**, except for one strain (AUSMDU00017262, Acc.: GCA_018602235.1) grouped in **G2-Sg3-Gn** (Figure 1). This suggests a clonal origin for its spread among **G2-Sg2-Pr**, with its initial entry possibly being a rare event in a common ancestor of this subgroup. The absence of SaPI2 carrying the *tst* gene in some strains of this subgroup may result from evolutionary pressures, such as the metabolic cost of maintenance, reduced selective advantage, or spontaneous excision (Ubeda et al., 2008). Strains without SaPI2 might also gain a fitness advantage in specific niches, with variability potentially arising from genetic drift or host-pathogen interactions (Baltrus, 2013).

McGavin et al. (2012) identified a deletion in the *trpD* gene in the MN8 strain, which explains the known tryptophan auxotrophy of mTSS strains and may contribute to the vaginal tropism of *tst*-carrying strains. Most strains (87.9%; *n* = 247/281) in **G2-Sg2-Pr** possess the same *trpD* allele as the MN8 strains, reinforcing the notion that this deletion has enhanced the host adaptability of strains of this subgroup.

Intriguingly, none of the strains from this subgroup carry PVL phages. Previous studies using the *agr* knockout strain TB4, along with complemented and overexpressed mutants using pSK9067, have demonstrated that the Agr system serves as a negative regulator of *tarM*, which encodes a glycosyltransferase responsible for the α-N-acetylglucosamine modification of the major *S. aureus* phage receptor, wall teichoic acids (WTA) (Yang et al., 2023). In fact, most WTA in *S. aureus* are adorned with β-N-acetylglucosamine by the TarS enzyme (Mistretta et al., 2019). Consequently, inhibition of the Agr system can hinder infections by bacteriophages that require a higher proportion of β-N-acetylglucosamine (Yang et al., 2023). Additionally, they showed that different ST types, including ST30 (strain MN8), were equally infected by the phage tested, suggesting the presence of similar phage receptors. Therefore, it is reasonable to suppose that the *agrC* mutation might render **G2-Sg2-Pr** strains resistant to PVL bacteriophages. An interesting future approach would be to test this hypothesis by complementing *agr*-RNAIII in an *agrC* mutant **G2-Sg2-Pr** strain to assess PVL phage infection. Remarkably, the only *tst*-positive strain outside the **G2-Sg2-Pr** cluster was found in the **G2-Sg3-Gn**. Unlike the *tst*-positive strains from **G2-Sg2-Pr**, which do not have *lukSF* genes and show a mutated *agrC* allele, this strain also carries the *lukSF* genes for producing PVL. Thus, we analyzed the *agrC* of this isolate and found a WT allele, which aligns with the premise above. It is well known that Agr inhibition decreases bacterial virulence in abscess model. Despite this, dysfunctional Agr strains can still cause severe invasive infections, including bloodstream infections (BSI). Moreover, this condition may confer advantages to hospital pathogens, as Agr impairment is associated with enhanced bacterial intracellular survival and persistent bacteremia (Das et al., 2016).

The prevalence of ST30-MSSA in **G2-Sg2-Pr**, particularly among the vulnerable age group of children under 2 years old, raises concerns due to their increased risk for *S. aureus* pneumonia (Neto et al., 2020). This association should raise important concerns within the medical community in countries where these clones are prevalent. Studies also indicate silent dissemination of *tst*-positive MSSA-ST30 in ICU patients and staff in Greece, posing a risk of hospital transmission and severe infections (Papadimitriou-Olivgeris et al., 2017). Additionally, research has shown a high prevalence of ST30-MSSA among children and infants (Achermann et al., 2015; Liang et al., 2021). This silent spread results from the focus of most epidemiological surveillance on identifying MRSA strains in hospitals.

Another notable aspect of this subgroup is the prevalence of the *sea* gene and the decrease of *chp* compared to **G1-Pk**, **G2-Sg2-Yw**, and **G2-Sg3-Gn**. The increased presence of IEC type-A (46.3%; *n* = 130/281), carrying *sea*, as the most prevalent type in this group is relevant, especially considering that IEC-B is generally more prevalent in *S. aureus* (Ahmadrajabi et al., 2017; Figure 4). Strains causing BSI that carry both *sea* and *tst* have been reported (Nienaber et al., 2011). Furthermore, studies using rabbit models of tampon-associated vaginal infections found that strains with both *tst+/sea +* exhibited greater virulence, indicating that the combination may enhance the pathogenic potential of these ST30 strains (Nienaber et al., 2011).

Moreover, 91.8% (*n* = 258/281) of genomes in this group lack the toxin-antitoxin system *toxM*, consistent with the findings of McGavin et al. (2012) regarding its absence in some CC30 genomes. Thus, it is possible that this absence may relate to a niche adaptation of this subgroup (McGavin et al., 2012). SaPI4, which lacks known virulence genes, was detected only in this subgroup. The genomes in this subgroup harbor a newly identified conjugative transposon (designated here as Tn*1421*), which carries a transposase of the IS*Sau5* and two ORFs annotated as staphylococcal secretory antigen-like (SsaA-like proteins) (Supplementary Table S1). SsaA is a peptidoglycan hydrolase known to be highly immunogenic. In *S. epidermidis,* SsaA has been linked to exacerbating nosocomial infections (Lang et al., 2000). Global gene expression studies have linked the *S. aureus ssaA* gene to biofilm production, suggesting that SsaA-like proteins may serve as virulence factors contributing to the success of these strains (Resch et al., 2005).

Another MGE, possibly an integrative plasmid of about 24 kb carrying two IS*1272* insertions, was specific to this ST30 subgroup. This IS is typically found in SCC*mec*IV, serving as a biomarker for differentiating SCC*mec* types (Boye et al., 2007). The presence of IS*1272* insertions outside SCC*mec* may lead to false positives for SCC*mec*IV (Viana et al., 2022). This MGE also contains genes associated with tolerance to cadmium, zinc, mercury, and arsenic, as well as genes encoding copper-transporting ATPase (*copB*) and multicopper oxidase (*mco*), which are typically mobilized together. Only certain *S. aureus* strains carry *copB*-*mco*, primarily those in CC22, CC30, and CC398 (Zapotoczna et al., 2018). These genes have been shown to enhance bacterial growth under copper stress and improve survival in macrophages and human blood (Zapotoczna et al., 2018), indicating that the entry of this MGE could significantly influence the evolution, spread, and pathogenesis of TSST-1-producing strains.

A unique insertion of IS*Sau2* was reported by McGavin et al. (2012) downstream of *saeRS* in the MN8 strain, which they speculated could disrupt the transcriptional terminator of *saeRS*, increasing gene expression and compensating for *agrC* mutations. However, in our study, this insertion was sporadic among ST30 isolates, predominantly occurring in **G2-Sg2-Pr** (7.1%; *n* = 20/281) compared to other ST30 strains (1.9%; *n* = 5/260), suggesting a rare and convergent evolutionary event, as they independently acquire this element. This subgroup exhibits a similar antimicrobial susceptibility pattern to other **G2** subgroups, except for methicillin, as most isolates are MSSA, and a higher frequency of *ermA* gene (40.2%; *n* = 113/281) (Supplementary Figure S2), the most common non-β-lactam antibiotic resistance gene reported among CC30 strains (Goudarzi et al., 2020; Viana et al., 2021).

#### 3.3.3 The evolutionary landscape of G2-Sg3-Gn

This subgroup includes 14.2% of the genomes analyzed (*n* = 77/541), primarily from Brazil (50.6%; *n* = 39/77), and several European countries (35.1%; *n* = 27/77). Of the 42 Brazilian ST30 genomes analyzed, only three clustered in **G2-Sg2-Pr**, while all others, including the Brazilian strains sequenced by our team, were MRSA and grouped in **G2-Sg3-Gn**. These strains account for 18.2% of CC30-SCC*mec*IV (*n* = 109) in a collection of 600 MRSA clinical strains isolated between 2014 and 2017 in Rio de Janeiro, originating from various clinical sources, including respiratory secretions, BSI, SSTI, and nasal swabs (Viana et al., 2021). The genome sizes of the 37 strains sequenced by us ranged from 2,792,580 bp to 2,963,657 bp, with GC content between 32.6 and 32.8%. The number of detected ORFs varied from 2,668 to 2,690, with most isolates carrying SCC*mec* type IVa and exhibiting variable *spa* types (Supplementary Table S1).

The SraP-D polymorphism, located in a variable region of 1916 bp, serves as the cladistic biomarker of this subgroup (Figure 5). There is also significant diversity in *spa* types, with t021 (24.7%; *n* = 19/77) and t318 (11.7%; *n* = 9/77) being the most prevalent (Table 1). Most genomes in this subgroup are MRSA (83.1%; *n* = 64/77), primarily associated with subtypes IVa (64.1%; *n* = 41/64) and IVc (17.2%; *n* = 11/64) of SCC*mec* (Table 1). Among the 68 strains with isolation source reported, mostly were from SSTIs (38.2%; *n* = 26/68) and colonization sites (33.8%; *n* = 23/68). PVL genes were detected in most strains (85.7%; *n* = 66/77) from haplotypes H2b (69.7%; *n* = 46/66) and H1a (30.3%; *n* = 20/66) (Table 2). However, O’Hara et al. (2008) associated haplotype R with MRSA and H with MSSA strains. This discrepancy could be due to the absence of ST30 strains in their study. They also indicated the haplotype H2 as the likely ancestor of H3 and H1 variants. In our study, PVL-H1a haplotype was detected in the more terminal subgroups of **G2**. Most PVL-positive strains from **G2-Sg3-Gn** (53.0%; *n* = 35/66) carry the PVL phage φ5967PVL (Table 2), which is considered a PVL phage likely not descended from earlier bacteriophages such as φPVL108 (Zhang et al., 2011).

IEC-B predominates in this subgroup (64.9%; *n* = 50/77), albeit at a lower frequency than in **G2-Sg1-Yw**, where nearly all genomes possess this IEC (Figure 4). IEC-A is present in 20.8% (*n* = 16/77) of **G2-Sg3-Gn** genomes, indicating the presence of the *sea* gene. The *v*Saα of this subgroup shows greater similarity to **G2** subgroups than to **G1-Pk**, which carries only the *lpl4* gene (Figure 3). In this subgroup, the *mecA* gene was found in 83.1% (*n* = 64/77) of genomes. Besides *mecA*, the *dfrG* gene was identified in 33.8% (*n* = 26/77) of genomes in this subgroup, excluding the Brazilian strains (Supplementary Table S2). This gene encodes dihydrofolate reductase which confers trimethoprim resistance. This is concerning, as sulfamethoxazole-trimethoprim is often recommended for treating uncomplicated skin infections, including those caused by CA-MRSA (Esposito et al., 2017).

**Figure 8:**
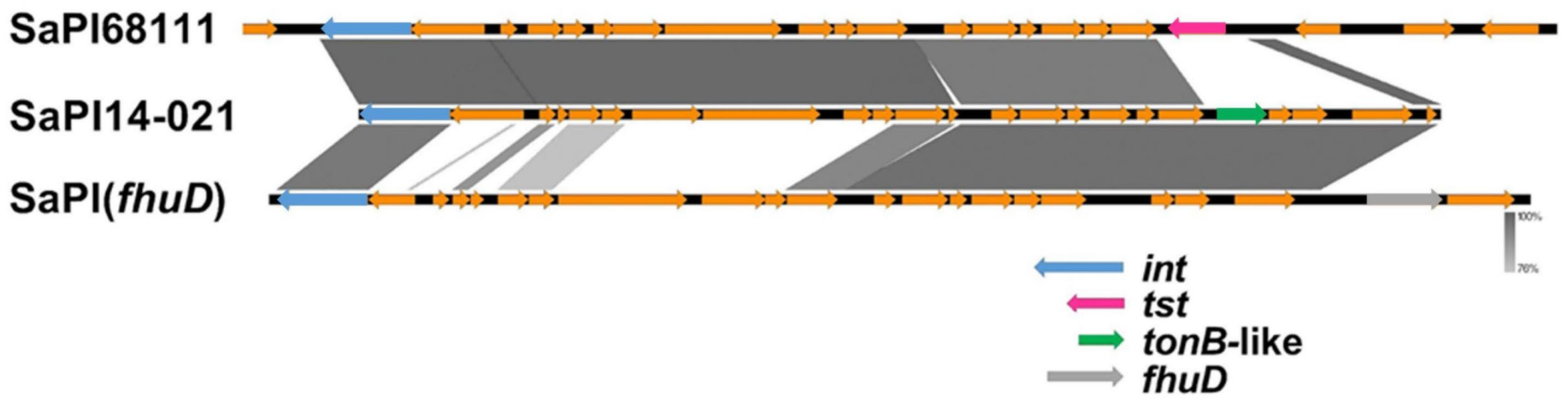
Comparative genomic alignment between SaPI-14-021, SaPI68111, and SaPI (*fhuD*). SaPI, staphylococcal pathogenicity island; *fhuD*, ferrichrome transport permease encoding gene; *tst*, toxic-shock toxin-1 encoding gene; *tonB*, TonB protein-encoding gene; *int*, integrase.

Only the genomes of **G2-Sg3-Gn** carried a novel SaPI, designated here as SaPI-14-021. This SaPI is located between positions 798,755 and 813,890 in the ST30-MRSA strain CR14-021 (Acc.: GCA_021012415.1), consisting of 26 coding regions. It integrates into an *att* site (TCCCGCCGTCTCCAT), identical to that of SaPIm4, and shares the same integrase sequence (Novick and Subedi, 2007). SaPI-14-021 shares 75% nucleotide identity with the SaPI (*fhuD*) (Khokhlova et al., 2015) and 79% with SaPI68111 (Li et al., 2011), suggesting it could result from a recombination of these two elements (Figure 8).

**Figure 9:**
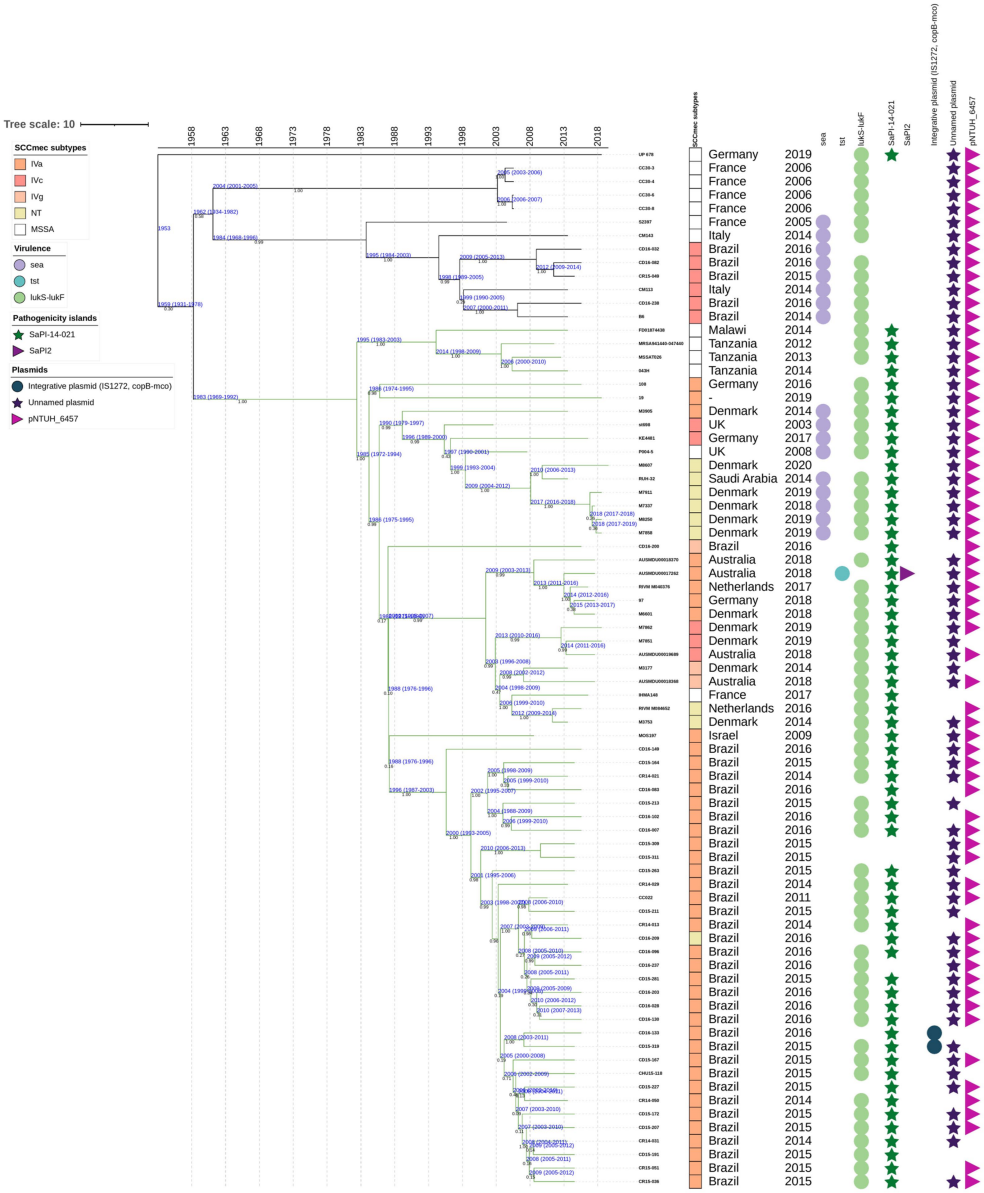
Time-calibrated Bayesian phylogenetic tree for the isolates included in the group 2, subgroup 3, green (**G2-Sg3-Gn**) obtained from 77 core genome alignments. Values in black indicate the posterior probability for each node. Values in blue indicate the age in years and the 95% highest probability density interval (HPDI) for each group. The maximum clade credibility tree was estimated using an uncorrelated log-normal relaxed clock model, with a random starting tree, and a coalescent constant tree prior. Green branches represent the SaPI-14-021 in international and Brazilian isolates. Rectangles represent the staphylococcal cassette chromosome *mec* (SCC*mec*) subtypes, depicted in different colors. Circles indicate the presence of specific genes: *sea* (encoding enterotoxin A), *tst* (encoding toxic-shock syndrome toxin-1), and *lukS-lukF* (encoding Panton-Valentine leukocidin). Pathogenicity islands SaPI-14-021 are shown as green stars, and SaPI2 as purple triangles. Plasmids are also illustrated: an integrative plasmid carrying two copies of IS*1272* and the *cop*-*mco* genes in blue circles; an unnamed plasmid in dark purple stars; and the pNTHU_6457 plasmid in a light-purple right-pointing triangle.

Notably, SaPI-14-021 lacks the *fhuD* gene, a key virulence marker of SaPI (*fhuD*), and instead contains an ORF that encodes a putative ferric-siderophore transport system protein (*tonB-*like) with no similarity with *fhuD*. This ORF was annotated using RASTtk (BV-BRC v. 3.39.10).^3^ Mosaicism involving pathogenicity islands is a well-documented phenomenon. Uehara et al. (2019) reported a mosaic pathogenicity island named SaPISaitama2, which includes the *tst*, *sec*, and *sel* genes, originating from the recombination of SaPIm4 and SaPIm1. Similarly, Iwao et al. (2012) described the SaPIj50 island as a mosaic of SaPIm4, PI T0131, and SaPIm1, carrying the *tst*, *sec*, *sel*, and *bla* genes. SaPI-14-021 was found in most strains in **G2-Sg3-Gn** (80.5%; *n* = 62/77), particularly in a more terminal subset (Figure 9). A search of the NCBI database revealed its presence also in the genomes of strain 13420, an ST30-SCC*mec*IV vancomycin-intermediate strain from Brazil (2009) (Chamon et al., 2020), and strain ER09001.3 [ST1472-(CC30)-SCC*mec*IV], isolated in the USA in 2017 (Caban et al., 2020).

SaPI-14-021 likely entered a basal ancestor of this subgroup around 1953. In the more terminal subset of **G2-Sg3-Gn**, it was present in nearly all strains, except for three, which share a more recent ancestor with the other strains in this subset, dating back to 1983 (95% HPDI 1969–1992) (Figure 9). Limited genome sequencing prevented a more comprehensive tracing of the evolutionary history of these strains. However, tree analysis suggests that the Brazilian strains likely originated in Europe, as the more basal genomes were from European countries (Figure 9). The tree structure also suggests that the island was likely acquired by the **G2-Sg3-Gn** genomes only once, was lost in some strains, but became highly conserved within the subset of Brazilian strains. Additionally, the most basal genome in the **G2-Sg3-Gn** cluster, UP678, also carries this island in the same location as the Brazilian strain CR14-021, further supporting this conclusion (Figure 9).

Another notable finding specific to this subgroup is the presence of a cadmium resistance plasmid, pNTUH_6457, found in 85.7% (*n* = 66/77) of strains (Acc.: LC377539, DDBJ database). Interestingly, this plasmid differs from the one associated with cadmium resistance in group **G1-Pk**, indicating a convergent pattern for cadmium resistance by distinct MGE. Additionally, an unnamed plasmid, present in 42.9% (*n* = 33/77) of these genomes—primarily in the second subset of strains that includes Brazilian isolates—carries 18 ORFs, including one coding for a penicillin-binding protein, one homologous to the *mecR1* gene, and another coding for an efflux ABC transporter. This may indicate a potential link between resistance and fitness/virulence, as efflux systems have been shown to be crucial for virulence and bacterial adaptability in various bacterial species (Olivares Pacheco et al., 2017; Wang-Kan et al., 2017; Gaurav et al., 2023).

## 4 Study limitation

While our collection includes strains from various regions worldwide and clinical materials isolated between 1990 and 2002, it may not fully represent the global MSSA and MRSA ST30 populations. This limitation arises primarily because many sequenced genomes are from more developed countries, where MRSA typically receives more attention than MSSA. Additionally, other uncontrollable variables may have influenced our sample selection. Despite conducting extensive manual curation of raw genomes, some annotated data may still be affected. Our analysis primarily focused on the presence or absence of genes and mobile genetic elements (MGEs), which may have led to the exclusion of significant evolutionary events, such as SNPs affecting gene regulators or other critical genes involved in bacterial host adaptation and virulence. Furthermore, we did not perform laboratory studies to confirm the impact of the identified events on bacterial pathogenicity. Nevertheless, it is important to emphasize that most virulence genes associated with significant evolutionary events in this study are well-established factors. Their roles in bacterial pathogenicity have been clearly demonstrated by our colleagues using knockout mutants, as described in the Results and Discussion section. Another potential limitation of our study was the use of a limited number of genomes for the divergence time analysis, which could affect the reliability of the timeline and reduce the precision of temporal inferences. However, the genomes included in the BEAST analysis were carefully selected to represent the major groups, subgroups, and subsets identified in the core genome phylogeny, ensuring that the most relevant diversity and evolutionary history were captured while maintaining computational feasibility for the time-calibrated analysis. Additionally, for these analyses, we used assembled genomes, focusing on high-quality assemblies that meet stringent criteria. While the analysis centered on conserved genomic regions, localized errors in assemblies may still affect the accuracy of chronological estimates. To minimize their impact, we focused on coding regions, selected appropriate evolutionary models, and filtered polymorphic sites.

## 5 Conclusion

This study provides a comprehensive analysis of ST30 evolution by examining over 500 genomes, including of contemporary MSSA and MRSA strains that emerged in the 21st century. The phylogenetic analysis reveals that SWP strains cluster with contemporary ST30 strains circulating globally. This cluster diverged from the PT80/81 strains around 1868 (95% HPDI: 1772–1934). It is possible that the absence of most *lpl* genes in strains related to PT80/81, as revealed in this study, may have at least partially limited their pandemic potential and global spread, likely explaining their absence among contemporary MRSA and MSSA ST30 strains. We found that ST30 MRSA isolates, circulating in both hospital and community settings in the 21st century, do not share a close relationship with PT80/81 strains but retain wild-type alleles for *agrC*, *hla*, and *isdH*. In contrast, all MSSA *tst*-positive strains analyzed in this study, including those involved in severe mTSS, exhibit mutated alleles. Therefore, while some authors have suggested that these mutations decrease pathogenicity and may confine strains to hospital infections, this hypothesis requires further validation. We found that most ST30 strains carrying the *tst* gene, associated with TSST-1, are MSSA and about half also possess the *sea* gene—an association likely contributing to the increased virulence of this MSSA clone. Additionally, these strains carry the *copB*-*mco* genes, which enhance not only survival under copper stress but also increase survival in macrophages and blood. We showed that during ST30 evolution, these strains acquired a considerable number of MGEs, many of which harbor well-known virulence factors. This may reflect the specialization of this MSSA strain toward becoming a well-adapted TSST-1 pathogen. Since most attention has been focused on MRSA isolates, MSSA *tst*-positive strains may have been overlooked, despite their potential to cause severe infections in hospitals where they circulate. Additionally, we show that the SraP polymorphism is apomorphic, and PCR-based approaches could be designed for the phylogenetic classification of ST30 strains. The Brazilian MRSA ST30 strains cluster in a more terminal subgroup of the phylogenetic group 2, while the Argentinean strains are grouped in a more basal subgroup, despite the geographic proximity of these countries. The Brazilian strains, which likely originated in Europe, have acquired a new SaPI-14-021, and ongoing studies aim to uncover its role in bacterial virulence and pathogenesis. Finally, our phylogenetic and time-calibrated analyses revealed a continuous intercontinental flow of **G2** strains, marked by their intense global dissemination. This pattern likely reflects the high prevalence of ST30 infections in healthy, non-hospitalized individuals, enabling their widespread distribution.

## Data Availability

The datasets presented in this study can be found in online repositories. The names of the repository/repositories and accession number(s) can be found in the article/Supplementary material.

## Footnotes

^1^ https://quast.sourceforge.net/

^2^ https://cge.food.dtu.dk/services/

^3^ https://www.bv-brc.org/view
