# Supplementary material for: Decoding the evolutionary history of ST30 Staphylococcus aureus: insights into a potentially silent MSSA bloodstream pathogen
Source: Front Microbiol. 2025 Apr 9;16:1522747. doi: 10.3389/fmicb.2025.1522747 (PMC12014664; doi:10.3389/fmicb.2025.1522747)
Supplement: Supplementary file 1 [file Data_Sheet_1.DOCX]

**Legend of Supplementary Figure S1. A** maximum-likelihood phylogenetic (branch length phylogeny) was constructed, including 541 genomes classified as ST30 MSSA or MRSA. Phylogenetic groups were defined based on tree topology, with monophyletic group 1, marked in pink (**G1-Pk**), and group 2 (**G2**), which is further divided into three subgroups: subgroup 1 (Sg1) in yellow (Yw) and designated as **G2-Sg1-Yw**; subgroup 2 (Sg2) in purple (Pr) and named **G2-Sg2-Pr**; and subgroup 3 (Sg3) in green (Gn) and referred to as **G2-Sg3-Gn**. The outer ring of the tree indicates the staphylococcal cassette chromosome *mec* (SCC*mec*) subtypes. Circles display the presence of specific virulence genes: *sea* (encoding enterotoxin A) in purple, *tst* (encoding toxic-shock syndrome toxin-1) in light blue, and *lukS-lukF* (encoding Panton-Valentine leukocidin) in light green. Stars represent genomes of the main *S. aureus* historical groups: Phage-Type 80/81 (PT80/81) in red, Southwest Pacific clone (SWP) in dark green, and menstrual toxic shock syndrome (mTSS) in dark blue. Pink squares represent the presence of Pathogenic Island SaPITokyo11212, purple triangles pointing rich represent SaPI2 and light-purple triangles SaPI4. Brown squares represent the presence of an unnamed plasmid carrying resistance genes for cadmium and macrolide resistance. Blue circles indicate an integrative plasmid containing insertion IS*1277*, the copper-transporting ATPase gene (*copB*), and the multicopper oxidase gene (mco). The purple star marks the unnamed plasmid detected in this study, while the light-purple right-pointing triangle represents pNTHU_6457, and the dark-pink left-pointing triangle represents plasmid Tn*1421*. Genome numbers highlighted in red denote those sequenced in this study.
