# Supplementary material for: Decoding the evolutionary history of ST30 Staphylococcus aureus: insights into a potentially silent MSSA bloodstream pathogen
Source: Front Microbiol. 2025 Apr 9;16:1522747. doi: 10.3389/fmicb.2025.1522747 (PMC12014664; doi:10.3389/fmicb.2025.1522747)
Supplement: Supplementary file 2 [file Data_Sheet_2.DOCX]

**Supplementary File S1.** Complete Materials and Methods Section.

**Genome selection.** Four hundred forty-one genomes were selected from a database containing a total of 3,015 genomes belonging to ST30 downloaded from NCBI on June 6, 2023, based on the regional diversity of the isolates and the availability of important information such as the isolation year, country, clinical origin/source, and genome quality (Supplementary Table S1). Additionally, 63 ST30 sequences from the Sequence Read Archive (SRA, NCBI) were added to include reference strains of phage-type 80/81 (DeLeo et al*.*, 2011, Rountree, 1958), the Southwest Pacific clone; SWP (McAdam et al., 2012), strain MN8, a typical representative of menstrual toxic shock syndrome (mTSS) (Reingold et al., 1982), and genomes of more contemporary strains from Argentina (Di Gregorio et al., 2021), Japan (Zuo *et a*l., 2021), and the Philippines (Masim et al*.*, 2021). Additionally, 37 genomes from Brazil sequenced by us were included (36 ST30 and one ST4279). The ST4279 genome of the MRSA strain CD15-309 differed from ST30 by only one nucleotide in the *glpF* gene. Supplementary Table S1 lists the NCBI accession (Acc.) and other important information concerning the 541 genomes included in this study. Molecular typing of the genomes was performed using MLST, accessible at https://github.com/tseemann/mlst, staphopia-SCCmec (Petit and Read, 2018) and spaTyper, available at https://github.com/HCGB-IGTP/spaTyper.

**Genome sequencing and assembly.** The MRSA strains sequenced here (one per patient) were nearly 30% of the CC30-MRSA detected in a collection of 600 MRSA clinical isolates of different lineages, isolated in 2014-2016 and obtained from hospitalized patients in Rio de Janeiro (Viana et al., 2021). Genomic DNA was obtained using the Wizard Genomic Kit as recommended by the manufacturer (PROMEGA, Madison, WI, USA), except that bacterial cells were suspended in TE buffer at pH 7.3 (10 µM Tris-HCl and 1 µM EDTA) and treated with lysostaphin (10 U/mL) prior to the addition of the lysis solution. Whole genome sequencing (WGS) was performed using paired-end sequencing and 300 cycles on the Illumina MiSeq. The library was constructed using the standard protocol of the Nextera DNA Flex Library Prep Kit as recommended by the manufacturer (Illumina, San Diego, CA, USA). Raw files (fastq) were trimmed using the BBDuk version 38.84 plugin of Geneious Prime version 2023.2.1 (Biomatters, Auckland, NZ) (parameters: trim all TruSeq, Nextera, and PhiX adapters; trim right end; trim low quality at both ends, with a minimum quality of 20; discard reads shorter than 20 bp). Normalization was performed using the BBNorm version 38.84 plugin of Geneious Prime (parameters: default sensitivity for error correction and normalization with a target coverage level of 40x, a minimum depth of 6x and a K-mer size of 31). Finally, de novo assembly was performed using Velvet version 1.2.10 hosted on the Geneious Prime platform (parameters: 27 k-mer length and minimum contig length of 200nt). Alternatively, when necessary, genomes were assembled into a contig using the Map to Reference tool available on Geneious Prime using trimmed and normalized files. The quality of the assembled genomes was assessed using QUAST v.5.2.0 (Gurevich et al., 2013), Only genomes meeting the following quality criteria were included: total length of 2.7 million bp ± 500 kb, GC content of 32.7% ± 1%, and a maximum of 200 N/100 kb. In addition, the preservation of repetitive informative sequences was validated using Mobile Element Finder v1.0.3 (accessible at https://cge.food.dtu.dk/services/ MobileElementFinder/).

One ST30 genome from Brazil (strain CR14-021; Acc. GCA_021012415.1) was fully assembled (in one contig) using the Map to Reference tool (reference genome strain Newman; Acc. AP009351) by a double assembly approach: de novo assembly performed using Velvet, and the referenced assembly, accomplished using the Map to Reference tool on Geneious Prime aligned using MAUVE (Darling et al., 2004). The consensus sequence was considered the fully assembled genome of strain CR14-021. General annotation and ORF predictions were automatically performed by the Geneious platform.

**Phylogenetic analysis and annotation.** The phylogenetic tree was constructed using 541 ST30 sequences. These sequences were annotated with Prokka v.1.14.6, and aligned with Roary v.3.13.0 using MAFFT, including only genes present in ≥99% of the genomes, ensuring high-quality alignments. Both Prokka and Roary are accessible at https://github.com/tseemann/prokka and https://github.com/sanger-pathogens/roary, respectively. The maximum likelihood (ML) tree was inferred using RAxML v.8.2.12, available at https://github.com/stamatak/standard-RAxML, with the GTR-CAT evolutionary substitution model and 100 bootstrap replicates. The tree was rooted using outgroup genomes from strains clustered in a more basal group, marked in pink in Figure 1. The Interactive Tree of Life (iTOL) v.6.9.1 tool, accessible at https://itol.embl.de was used for tree visualization and editing.

**Time-scaled evolutionary analysis**. A Bayesian phylogenetic analysis was performed with 61 genome representatives of all groups and subgroups according to the architecture of the phylogenetic tree composed of 541 genomes. Another Bayesian phylogeny was accomplished with all genome sequences of the subgroup that clusters the Brazilian strains sequenced here. The genomes included in the BEAST analysis were carefully selected to ensure they represent the major clades and subgroups identified in the core genome tree constructed from the 541 genomes. This selection strategy allowed us to capture the most relevant diversity and evolutionary history, while also ensuring computational feasibility for the time-calibrated analysis. By choosing a representative subset, we balanced the need for comprehensive data with the practical constraints of computational resources. Genomes showing signs of poor assembly quality, such as low completeness or high contamination, were excluded. The use of assemblies is justified by the study’s objective of inferring macro-evolutionary events. The following measures were taken to ensure robust results: (i) Only coding regions were included to minimize noise from poorly aligned areas. (ii) Evolutionary models were carefully selected to ensure reliable inferences. (iii) Recombination effects were minimized by focusing on conserved regions and filtering polymorphic sites using ClonaFrameML. The core genome alignment was accomplished using Roary. The ML tree was constructed with RAxML with the General Time Reversible (GTR), Gamma model, and 100 bootstraps. ClonalFrameML v.1.13, available at https://github.com/xavierdidelot/ClonalFrameML, filtered polymorphic sites that could affect the time-scaled analysis. Bayesian phylogenetic analysis was carried out by BEAST v.1.10.4 (Suchard et al*.*, 2018) with the HKY model of nucleotide substitution with estimated base frequencies, Gamma site heterogeneity, and an uncorrelated log-normal relaxed clock model, with a random start tree and a coalescent constant size tree prior. The substitution rate prior value adopted was 1.5 x 10^-6^ as the ucld.mean parameter, allowing the distribution to vary between 1.0 x 10^-10^ and 1.0 x 10^-3^. The final substitution rate inferred for the BEAST with representative genomes of all groups and subgroups was 3.5 x 10^-6^ (95% HPD interval of 1.11 x 10^-5^ and 4.11 x 10^-6^), and for the BEAST with the subgroup with Brazilian genomes was 2.30 x 10^-6^ (with a 95% HPD interval of 1.55 x 10^-6^ and 3.11 x 10^-6^); values similar to those found by Duchêne and colleagues (2016). We ran two independent Markov chain Monte Carlo (MCMC) chains for 200 million iterations each run, discarding the first 10% as burn-in. MCMC chains were visualized with Tracer v.1.7.1 and the maximum clade credibility tree was calculated with TreeAnnotator v.1.10.4. The resulting trees were visualized and annotated with iToL v.6.9.1.

**Virulence, resistance, and mobile genetic elements.** For all 541 genomes, virulence content was analyzed with the BLASTn command line (https://www. ncbi.nlm.nih.gov/books/NBK569861 accessed on September 27, 2024) and the Virulence Finder Database (VFDB) database (Chen et al*.*, 2005) with the MRSA252 genome [ST36(CC30)-SCC*me*cII, GenBank accession number (Acc): GCA_009460765.1] used as a query. Some sequences were manually annotated using the UniProt database. Due to the paralogs that could lead to annotation errors of *lukSF*-PVL, the reference sequences of these genes were downloaded as contiguous sequences (strain 13420; Acc: NZ_CP021141.1, nucleotide (nt) position 1,5198,664 - 1,520,581). For all searches, BLASTn results were considered positive if hits exceeded 90% coverage and 95% identity.

Previously described gene alleles proposed for ST30 classification or containing important mutations, such as *agrC*, *hla* (De Leo et al., 2011), *isdH* (McGavin et al., 2012), and *saeS* (Ramundo et al., 2016), were also searched using the BLASTn command line tool. To perform allelic classification of *agrC*, *hla,* *isdH,* and *saeS* genes both alleles (mutated; mut and wild-type; WT) for each gene were used as queries [*agrC_*WT and *agr_*mut (Gly55Arg)*; saeS_*WT and *saeS_*SKT (N227S, E268K and S351T); *hla_*WT and *hla_*mut (stop codon at residue 112 instead of Gln)*; isdh*_WT and *isdH_*mut (stop codon at residue 79 instead of Gln)], as previously described (DeLeo et al*.*, 2011; McGavin et al., 2012; Ramundo et al*.*, 2016). Parameters adopted were 90% nucleotide coverage and 100% identity. Lower identity results were manually inspected, and the higher identity match was used for classification.

Classical genomic islands of *S. aureus* were initially detected using specific genetic biomarkers present in the *v*Saα (*ssl2* and *ssl10*), *v*Saβ (*sen*, *seo,* and *sei*), *v*Saγ (*argF*, *murL,* and *hla*) and IEC type A-F (sea, *sak*, *chp*, *scn*) (Kuroda et al., 2001; Siboo et al., 2008; Aswani et al., 2019; van Wamel et al. 2016). Representative genomes of each phylogenetic group were then selected to localize these genomic islands using the "map to reference" tool of Geneious Prime Software and visual inspection (**G2-Pk**: strain 68-397, GenBank accession: GCA_000160375.1; **G2-Sg1-Yw**: strain CM92, accession: GCA_003237675.1; **G2-Sg2-Pr**: strain CFSA198, accession GCA_002124595.1; and **G2-Sg3-Gn**: GCA_900251255.1). The reference sequence of each genomic island and IEC was identified and analyzed in the 541 genomes using the BLAST command line with 95% identity and coverage. Variations in each island or IEC was also analyzed using MAUVE alignment software (version 2024.04) (Darling et al*.*, 2011).

The detection of staphylococcal pathogenic islands (SaPIs) 1 to 4 was performed by identifying key biomarkers specific to each island. SaPI1 was identified by the presence of *seb*, SaPI2 by *tst*, SaPI3 by *sec* and *sel*, and SaPI4 and SaPIbov by *guaA* and *guaB*. Further analysis involved extracting the open reading frames (ORFs) of each SaPI from representative genomes: COL (SaPI1, GenBank accession: JBGOFO000000000.1), MN8 (SaPI2 and SaPI4, accession: NZ_CM000952.1), and USA400 (SaPI3, accession: NZ_JAFFHW000000000.1) using the Map to Reference tool in Geneious Prime Software. The genetic context of each SaPI was manually inspected based on previous ORF annotations (McGavin et al., 2012). Finally, the reference sequence of each SaPI was identified and analyzed in the 541 genomes using the BLAST command line with 95% identity and coverage. For some analyses, sequence variation was also examined using MAUVE alignment software.

Antimicrobial resistance genes were detected using the CARD database with ABRicate, available at https://github.com/tseemann/abricate, with a minimum identity and coverage of 90%.

**Panton-Valentine phage prediction.** A region containing the *lukSF* genes plus 300-400 bp downstream and upstream of these genes was obtained from the nucleotide sequence of the PVL phage phiPVL2958 (Acc. Number: NC_011344.1), phiPVL5967 (Acc. Number: AP011955.1), phiPVL108 (Acc. Number: NC_008689.1), phiPVLCN125 (Acc. Number: NC_012784.1), phiSa2USA (Acc. Number: KX130855.1), phiSLT (Acc. Number: NC_002661.2) and phiTP310 (Acc. Number: EF462197.1) commonly found in ST30 strains (Boakes et al*.*, 2011; Chen et al*.*, 2013; Zhang et al*.,* 2011; Di Gregorio et al., 2021; Najafi Olya et al., 2021). The regions were used as queries to conduct BLASTn using all the genomes from this study. The PVL phage attributed to each genome was the sequence with an identity higher than 99.9% (≥90% coverage).

**Pangenomic analysis.** To assess important genomic changes on mobile genetic elements (MGEs) that occurred during the evolution of ST30-MSSA and ST30-MRSA strains we used an ORF-based pangenomic binary (1, 0) matrix approach as previously described (Esteves et al., 2023). The reference strains used were NCTC11561 (GCA_900458105.1), Sa-122 (GCA_028389215.1), MN8 (GCA_022163365.1), and CR14-021 (Acc.: GCA_021012415.1), representing all groups and subgroups revealed in the LM tree with 541 genomes. Volcano plots were generated and a cutoff of 50 was established for -log10 *p*-value for each pair of groups/subgroups analyzed. ORF sequences with the lowest *p*-values were localized in the reference genomes using Geneious Prime, Map to Reference tool.

***SraP* polymorphism typing**

Different *sraP* types were detected by manual inspection with Geneious Prime Software, one representative of each type was selected and the region containing these genes was obtained using the Extract tool of Geneious Prime, ORFs were manually annotated. Sequence variation in each island was analyzed using MAUVE alignment software (version 2024.04). The variable region of the *sraP* gene was extracted from representative strains from each group and subgroup. **G1-Pk**, *SraP*-A (strain 68-397, access number: GCA_000160375.1); **G2-Sg1-Yw**, *SraP*-B (strain CM92, access number: GCA_003237675.1); **G2-Sg2-Pr**, *SraP*-C (strains CFSA198, access number: GCA_002124595.1); **G2-Sg3-Gn**, *SraP*-D (strain M3177, access number: GCA_900251255.1).

**References**

Aswani, V., Najar, F., Pantrangi, M., Mau, B., Schwan, W. R., and Shukla, S. K. (2019). Virulence factor landscape of a *Staphylococcus aureus* sequence type 45 strain, MCRF184. *BMC Genomics* 20, 123. doi: 10.1186/s12864-018-5394-2

Boakes, E., Kearns, A. M., Ganner, M., Perry, C., Hill, R. L., and Ellington, M. J. (2011). Distinct bacteriophages encoding Panton-Valentine leukocidin (PVL) among international methicillin-resistant *Staphylococcus aureus* clones harboring PVL. *J. Clin. Microbiol*. 49, 684–692. doi: [10.1128/JCM.01917-10](https://doi.org/10.1128/JCM.01917-10)

Chen, L. (2004). VFDB: a reference database for bacterial virulence factors. *Nucleic Acids Res.* 33, D325–D328. doi: [10.1093/nar/gki008](https://doi.org/10.1093/nar/gki008)

Chen, L., Chavda, K. D., Solanki, M., Mediavilla, J. R., Mathema, B., Schlievert, P. M., et al. (2013). Genetic variation among Panton-Valentine leukocidin-encoding bacteriophages in *Staphylococcus aureus* clonal complex 30 Strains. *J. Clin. Microbiol*. 51, 914–919. doi: [10.1128/JCM.03015-12](https://doi.org/10.1128/JCM.03015-12)

Darling, A. C. E., Mau, B., Blattner, F. R., and Perna, N. T. (2004). Mauve: multiple alignment of conserved genomic sequence with rearrangements. *Genome Res.* 14, 1394–1403. doi: [10.1101/gr.2289704](https://doi.org/10.1101/gr.2289704)

DeLeo, F. R., Kennedy, A. D., Chen, L., Wardenburg, J. B., Kobayashi, S. D., Mathema, B., et al. (2011). Molecular differentiation of historic phage-type 80/81 and contemporary epidemic *Staphylococcus aureus*. *Proc. Natl. Acad. Sci. U.S.A.* 108, 18091–18096. doi: [10.1073/pnas.1111084108](https://doi.org/10.1073/pnas.1111084108)

Di Gregorio, S., Haim, M. S., Vielma Vallenilla, J., Cohen, V., Rago, L., Gulone, L., et al. (2021). Genomic epidemiology of CC30 methicillin-resistant *Staphylococcus aureus* strains from Argentina reveals four major clades with distinctive genetic features. *mSphere* 6, e01297-20. doi: [10.1128/mSphere.01297-20](https://doi.org/10.1128/mSphere.01297-20)

Duchêne, S., Holt, K. E., Weill, F.-X., Le Hello, S., Hawkey, J., Edwards, D. J., et al. (2016). Genome-scale rates of evolutionary change in bacteria. *Microb. Genom.* 2. doi: [10.1099/mgen.0.000094](https://doi.org/10.1099/mgen.0.000094)

Esteves, M. A. C., Viana, A. S., Viçosa, G. N., Botelho, A. M. N., Moustafa, A. M., Mansoldo, F. R. P., et al. (2023). RdJ detection tests to identify a unique MRSA clone of ST105-SCC*mec*II lineage and its variants disseminated in the metropolitan region of Rio de Janeiro. *Front. Microbiol.* 14, 1275918. doi: 10.3389/fmicb.2023.1275918

Gurevich, A., Saveliev, V., Vyahhi, N., and Tesler, G. (2013). QUAST: quality assessment tool for genome assemblies. *Bioinformatics* 29, 1072–1075. doi: [10.1093/bioinformatics/btt086](https://doi.org/10.1093/bioinformatics/btt086)

Kuroda, M., Ohta, T., Uchiyama, I., Baba, T., Yuzawa, H., Kobayashi, I., et al. (2001). Whole genome sequencing of meticillin-resistant *Staphylococcus aureus*. *The Lancet* 357, 1225–1240. doi: [10.1016/S0140-6736(00)04403-2](https://doi.org/10.1016/S0140-6736(00)04403-2)

Masim, M., Argimon, S., Espiritu, H., Magbanua, M., Olorosa, A., Cohen, V., et al. (2021). Genomic surveillance of methicillin-resistant *Staphylococcus aureus* in the Philippines, 2013–2014. *WPSAR*. 12, 6–16. doi: [10.5365/wpsar.2020.11.1.004](https://doi.org/10.5365/wpsar.2020.11.1.004)

McAdam, P. R., Templeton, K. E., Edwards, G. F., Holden, M. T. G., Feil, E. J., Aanensen, D. M., et al. (2012). Molecular tracing of the emergence, adaptation, and transmission of hospital-associated methicillin-resistant *Staphylococcus aureus*. *Proc. Natl. Acad. Sci. U.S.A.* 109, 9107–9112. doi: [10.1073/pnas.1202869109](https://doi.org/10.1073/pnas.1202869109)

McGavin, M. J., Arsic, B., and Nickerson, N. N. (2012). Evolutionary blueprint for host- and niche-adaptation in *Staphylococcus aureus* clonal complex CC30. *Front. Cell. Inf. Microbio.* 2. doi: [10.3389/fcimb.2012.00048](https://doi.org/10.3389/fcimb.2012.00048)

Najafi Olya, Z., Najar-Peerayeh, S., Yadegar, A., and Bakhshi, B. (2021). Clonal diversity and genomic characterization of Panton-Valentine Leukocidin (PVL)-positive *Staphylococcus aureus* in Tehran, Iran. *BMC Infect. Dis*. 21, 372. doi: [10.1186/s12879-021-06060-4](https://doi.org/10.1186/s12879-021-06060-4)

Petit, R. A., and Read, T. D. (2018). *Staphylococcus aureus* viewed from the perspective of 40,000+ genomes. *PeerJ*. 6, e5261. doi: [10.7717/peerj.5261](https://doi.org/10.7717/peerj.5261)

Ramundo, M. S., Beltrame, C. O., Botelho, A. M. N., Coelho, L. R., Silva-Carvalho, M. C., Ferreira-Carvalho, B. T., et al. (2016). A unique SaeS allele overrides cell-density dependent expression of *saeR* and *lukSF*-PV in the ST30-SCCmecIV lineage of CA-MRSA. *Int. J. Med. Microbiol*. 306, 367–380. doi: [10.1016/j.ijmm.2016.05.001](https://doi.org/10.1016/j.ijmm.2016.05.001)

Reingold, A. L. (1982). Toxic shock syndrome surveillance in the United States, 1980 to 1981. *Ann. Intern. Med*. 96, 875. doi: [10.7326/0003-4819-96-6-875](https://doi.org/10.7326/0003-4819-96-6-875)

Rountree, P. M., and Beard, M. A. (1958). Further observations on infection with phage type 80 staphylococci in Australia. *Med. J. Aust*. 45, 789–795.

       Siboo, I. R., Chambers, H. F., and Sullam, P. M. (2005). Role of SraP, a serine-rich surface protein of *Staphylococcus aureus*, in binding to human platelets. *Infect. Immun.* 73, 2273–2280. doi: 10.1128/IAI.73.4.2273-2280.2005

Suchard, M. A., Lemey, P., Baele, G., Ayres, D. L., Drummond, A. J., and Rambaut, A. (2018). Bayesian phylogenetic and phylodynamic data integration using BEAST 1.10. *Virus Evolution* 4. doi: [10.1093/ve/vey016](https://doi.org/10.1093/ve/vey016)

Van Wamel, W. J. B., Rooijakkers, S. H. M., Ruyken, M., Van Kessel, K. P. M., and Van Strijp, J. A. G. (2006). The innate immune modulators Staphylococcal complement inhibitor and chemotaxis inhibitory protein of *Staphylococcus aureus* are located on β-Hemolysin-converting bacteriophages. *J. Bacteriol*. 188, 1310–1315. doi: [10.1128/JB.188.4.1310-1315.2006](https://doi.org/10.1128/JB.188.4.1310-1315.2006)

Viana, A. S., Nunes Botelho, A. M., Moustafa, A. M., Boge, C. L. K., Pires Ferreira, A. L., Da Silva Carvalho, M. C., et al. (2021). Multidrug-resistant methicillin-resistant *Staphylococcus aureus* associated with bacteremia and monocyte evasion, Rio de Janeiro, Brazil. *Emerg. Infect. Dis.* 27, 2825–2835. doi: [10.3201/eid2711.210097](https://doi.org/10.3201/eid2711.210097)

Zhang, M., Ito, T., Li, S., Jin, J., Takeuchi, F., Lauderdale, T.-L. Y., et al. (2011). Identification of the third type of PVL phage in ST59 methicillin-resistant *Staphylococcus aureus* (MRSA) strains: CA-MRSA strains worldwide might carry distinct PVL phages. *FEMS Microbiol. Lett*. 323, 20–28. doi: [10.1111/j.1574-6968.2011.02355.x](https://doi.org/10.1111/j.1574-6968.2011.02355.x)

Zuo, H., Uehara, Y., Lu, Y., Sasaki, T., and Hiramatsu, K. (2021). Genetic and phenotypic diversity of methicillin-resistant *Staphylococcus aureus* among Japanese inpatients in the early 1980s. *Sci. Rep*. 11, 5447. doi: [10.1038/s41598-021-84481-6](https://doi.org/10.1038/s41598-021-84481-6)
